# Integration of metabolomics and transcriptomics provides insights into the molecular mechanism of temporomandibular joint osteoarthritis

**DOI:** 10.1371/journal.pone.0301341

**Published:** 2024-05-16

**Authors:** Palati Tuerxun, Takkun Ng, Ke Zhao, Ping Zhu

**Affiliations:** 1 Hospital of Stomatology, Sun Yat-sen University, Guangzhou, Guangdong Province, China; 2 Guangdong Provincial Key Laboratory of Stomatology, Guangzhou, Guangdong Province, China; 3 Guanghua School of Stomatology, Sun Yat-sen University, Guangzhou, Guangdong Province, China; University of South Carolina, UNITED STATES

## Abstract

The deficiency of clinically specific biomarkers has made it difficult to achieve an accurate diagnosis of temporomandibular joint osteoarthritis (TMJ-OA) and the insufficient comprehension of the pathogenesis of the pathogenesis of TMJ-OA has posed challenges in advancing therapeutic measures. The combined use of metabolomics and transcriptomics technologies presents a highly effective method for identifying vital metabolic pathways and key genes in TMJ-OA patients. In this study, an analysis of synovial fluid untargeted metabolomics of 6 TMJ-OA groups and 6 temporomandibular joint reducible anterior disc displacement (TMJ-DD) groups was conducted using liquid and gas chromatography mass spectrometry (LC/GC-MS). The differential metabolites (DMs) between TMJ-OA and TMJ-DD groups were analyzed through multivariate analysis. Meanwhile, a transcriptomic dataset (GSE205389) was obtained from the GEO database to analyze the differential metabolism-related genes (DE-MTGs) between TMJ-OA and TMJ-DD groups. Finally, an integrated analysis of DMs and DE-MTGs was carried out to investigate the molecular mechanisms associated with TMJ-OA. The analysis revealed significant differences in the levels of 46 DMs between TMJ-OA and TMJ-DD groups, of which 3 metabolites (L-carnitine, taurine, and adenosine) were identified as potential biomarkers for TMJ-OA. Collectively, differential expression analysis identified 20 DE-MTGs. Furthermore, the integration of metabolomics and transcriptomics analysis revealed that the tricarboxylic acid (TCA) cycle, alanine, aspartate and glutamate metabolism, ferroptosis were significantly enriched. This study provides valuable insights into the metabolic abnormalities and associated pathogenic mechanisms, improving our understanding of TMJOA etiopathogenesis and facilitating potential target screening for therapeutic intervention.

## Introduction

Temporomandibular joint (TMJ) osteoarthritis (TMJ-OA) is characterized by the progressive and chronic deterioration of the articular cartilage and remodeling of the underlying bone structures, causing significant pain and functional limitations in the temporomandibular joint [1, 2]. The etiology of TMJ-OA is associated with multiple factors, including aging, gender, mechanical stress, oral parafunctional habits, and psychological stress [3]. In the current clinical practice, conservative treatment remains the primary therapeutic approach for TMJ OA, involving measures like joint protection, physical therapy, occlusal splints, and the administration of nonsteroidal anti-inflammatory drugs (NSAIDs) [4]. However, due to the uncertain etiology and pathogenesis, the current conservative therapeutic approaches for TMJ OA appear to be ineffective in curing this chronic condition [5]. Currently, there is an urgent need for the identification of novel biomarkers that can facilitate early diagnosis of TMJ-OA, with the aim of reversing the further progression of the TMJ-OA during its initial stages. Additionally, the discovery of new and effective therapeutic targets is extremely necessary to improve treatment strategies for patients with TMJ-OA.

The high concentration of metabolites in biological body fluids, including urine, plasma, and synovial fluid, represents a valuable source of biological information that can greatly contribute to our understanding of tissue metabolism [6]. During pathological conditions, changes in the concentrations of endogenous substances in biological fluids occur due to disease-related biochemical reactions [7]. Due to its capacity to directly reflect the biological processes of cartilage and other joint tissues, synovial fluid represents an invaluable specimen for conducting TMJ-OA studies with the ability to assess metabolite concentrations.

Metabolomics, which involves the comprehensive analysis of the entire spectrum of metabolites present in living organisms, serves as a powerful tool for helping to identify biomarkers and analyze metabolic pathways to provide insights into the pathological mechanisms of diseases and establish a robust clinical diagnosis model [8]. Alterations in the metabolite occur rapidly, providing a comprehensive representation of the biological response resulting from variations in the genome, transcriptome, and proteome [9]. Within an environment lacking adequate oxygen and glucose supply, cartilage engages in active metabolism, which presents a multitude of metabolic challenges to the cartilage itself [10]. Under pathophysiological conditions, cellular metabolism experiences significant disruption, resulting in an upregulation of anti-anabolic, pro-catabolic, and pro-inflammatory factors [11]. Previous studies have utilized metabolomic analysis of synovial fluid to explore the metabolic changes occurring in OA and to identify biomarkers that could be indicative of the condition [12, 13]. Transcriptomics provides a means to decipher the functional components of the genome and illuminate the disease-associated gene expression profiles across various biological pathways. However, the identification of metabolite and metabolism-related genes associated with metabolic pathways that potentially play a crucial role in initiating and advancing TMJ-OA is still unclear.

In this study, we examined metabolic changes in the synovial fluid of individuals with TMJ-OA and controls groups, and identified potential biomarkers that could be used for diagnosing TMJ-OA. Considering the direct involvement of synovial tissue in the pathogenesis of TMJ-OA [14], therefor we utilized transcriptomic data from this synovial tissue to gain a comprehensive understanding of the underlying pathological mechanisms driving TMJ-OA. In conclusion, we performed a comprehensive integrative analysis of metabolomics and transcriptomics to elucidate the altered metabolic pathways involved in TMJ-OA.

## Materials and methods

### Patient groups

Patients (mean age 40.08 ± 16.52, range 17–71 years), 1 man (mean age 20) and 11 women (mean age 41.91 ± 16.6) diagnosed with temporomandibular joint with disc displacement (TMJ-DD) or TMJ-OA were recruited from the Hospital of Stomatology at Sun Yat-sen University for this study. Participants were recruited based on specific inclusion criteria: 1) participants were enrolled based on their diagnosis of one or multiple temporomandibular disorders according to the RDC/TMD criteria [15]; 2) the absence of any signs or symptoms of systemic or local diseases that could imitate temporomandibular disorders (TMJ trauma, bilateral ailments, autoimmune diseases, rheumatoid arthritis, history of acute or chronic infections, severe endocrine disorders, cancers, and current use of anti-inflammatory medication) [15]. The diagnosis of patients with disc displacement and condylar cartilage destruction was confirmed through the utilization of magnetic resonance imaging (MRI) and cone-beam computed tomography (CBCT) [16]. The local ethics committee of hospital of stomatology at Sun Yat-sen University provided ethical approval (reference no. KQEC-2023-27-02) for the collection of human synovial fluid lavage in this study, and informed written consent was obtained from all subjects. All methods were performed in accordance with the relevant guidelines and regulations.

### Sample collection

The local ethics committee of hospital of stomatology at Sun Yat-sen University provided ethical approval (reference no. KQEC-2023-27-02) for the collection of human synovial fluid lavage in this study. Six TMJ-DD individuals (control group) and six TMJ-OA patients (experimental group) were recruited from the oral and maxillofacial surgery clinic at hospital of stomatology at Sun Yat-sen University for synovial fluid. Subsequently, a cross-shaped pressure was applied with a finger at approximately 1 cm in front of the ear to confirm the location and disinfection. Using a 5 mL syringe, 3 mL of 2% lidocaine was aspirated. Following confirmation of blood-free aspiration, anesthetic lavage was conducted, and 1 mL of joint cavity lavage fluid was obtained. The samples underwent centrifugation at 12,000 rpm for 15 minutes at 4°C. After centrifugation, the upper layer of the synovial fluid lavage was diligently collected and distributed into 0.5 mL per tube using 1.5 mL centrifuge tubes. The tubes were then stored in a -80°C freezer.

### Sample preparation

The samples stored at -80°C were thawed to room temperature. Incorporating 150 μL of the sample and 10 μL of L-2-chlorophenylalanine (0.3 mg/mL) dissolved in methanol as an internal standard, the mixture was transferred to a 1.5 mL centrifuge tubes. The tubes were then vortexed for 10 seconds to ensure thorough blending. After vortexing the mixture for 10 seconds, it was incubated at 4°C for 10 minutes, and then subjected to centrifugation at 13,000 rpm at 4°C for 15 minutes. The 150 μL supernatant was carefully transferred into a fresh vial and subjected to vacuum drying at room temperature. Equal volumes of the supernatant were combined to generate quality controls (QC). The resultant mixture was vortexed vigorously for 2 min and incubated at 37°C for 90 min. The samples were placed at ambient temperature for 30 min before liquid and gas chromatography mass spectrometry (LC/GC-MS) analysis.

### LC/GC-MS analysis

The 1μL of derivatized sample was injected into splitless mode an Agilent 7890B equipped with a DB-5MS fused-silica capillary column (30 m × 0.25 mm × 0.25 μm, Agilent J & W Scientific, Folsom, CA, USA) utilized to separate the derivatives. Helium (> 99.999%) was used as the carrier gas at a constant flow rate of 1 mL / min through the column. The injector temperature was maintained at 260°C. The initial oven temperature was held at 60°C for 0.5 min, ramped to 125°C at a rate of 8°C/min, and finally held at 305°C for 5 min [17, 18]. For the transfer line, a temperature of 150°C was selected, while the ion source was kept at 230°C. Ions were generated using a 70 eV electron beam operating at a current of 2.0 mA. The mass scanning range extended from m/z 45 to 800, with a scanning rate of 30 spectra per second.

### Differential metabolites between TMJ-OA and TMJ-DD

The raw data were imported into software Progenesis QI V2.3 (Nonlinear, Dynamics, Newcastle, UK), which performs peak detection, peak identification, characterization, peak alignment, wave filtering, and missing value interpolation. In each sample, all peak signal intensities were segmented and normalized according to the internal standards with RSD greater than 0.3. After data processing, the matrix was imported in RStudio software (Chicago, MA, USA)) to carry out principle component analysis (PCA) to observe the overall distribution among the samples and the stability of the whole analysis process. The orthogonal partial least-squares-discriminant analysis (OPLS-DA) was utilized to distinguish the metabolites that differ between groups. Variable importance of projection (VIP) values obtained from the OPLS-DA model were used to rank the overall contribution of each variable to group discrimination. Differential metabolites (DMs) were selected with VIP values greater than 1.0 and p-values less than 0.05. For the identification of key pathways associated to the DMs, the Kyoto Encyclopedia of Genes and Genomes database (KEGG) (http://www.genome.jp/kegg/) was employed. MetaboAnalyst 5.0 (https://genap.metaboanalyst.ca/MetaboAnalyst), a specialized software tool designed for the analysis and interpretation of metabolomic data, was used to perform key pathways analyses on the DMs.

### Differential metabolism-related genes between TMJ-OA and TMJ-DD

The mRNA expression profile datasets (GSE205389), including five individuals with TMJ-DD (control group) and five patients with TMJ-OA (experimental group), were obtained from the Gene Expression Omnibus (GEO) (https://www.ncbi.nlm.nih.gov/geo/) database. To ensure the pertinence and applicability of this dataset to our analysis, we conducted a comprehensive review of the demographic and clinical features as well as the inclusion criteria of the patients included in the GSE205389 dataset. The main objective of our study is to conduct a metabolomic analysis utilizing synovial fluid samples taken from patients suffering from osteoarthritis. Despite the variance in sample types (synovial fluid in our study versus synovial tissue in GSE205389), both datasets originate from individuals diagnosed with osteoarthritis, enabling a complementary analysis.

The “*limma*” package [19] for RStudio was utilized to identify differentially expressed genes (DEGs) between TMJ-OA and TMJ-DD in the GSE205389 datasets. The p-values <0.05 and log2-fold change (FC) | ≥ 1 were set as the cut-off criteria for DEGs identification. Metabolism-related genes (MTGs) were downloaded from the c2.cp.kegg.v7.0.symbols dataset in the MSigDB database. The differentially expressed metabolism-related genes (DE-MTGs) were obtained by intersecting the MTGs with the DEGs.

### Analysis of functional enrichment and pathways of DE-MTGs

The “*clusterProfiler*” package [20] in Bioconductor was used for Gene Ontology (GO) and KEGG pathway enrichment analysis to illustrate the functions of the DE-MTGs. Then, GO and KEGG pathway enrichment analyses were conducted. A significance level of p-values <0.05 was used as a cut-off standard.

### Integrative analysis of metabolomics and transcriptomics

In order to identify TMJ-OA-related biological function, the integration of DMs and DE-MTGs was performed using the OmicsNet module on MetaboAnalyst 5.0. Then, KEGG pathway enrichment analyses were conducted.

### Statistical analysis

RStudio version 4.0.0 was applied for bioinformatics analysis. We compared the differences between two groups with an unpaired Student’s t test, and the results of all statistical analyses are presented as the means ± standard deviations (SDs). For all the statistical test results, p values < 0.05 were considered statistically significant.

## Results

### Multivariate PCA and OPLS-DA analysis

**Fig 1:**
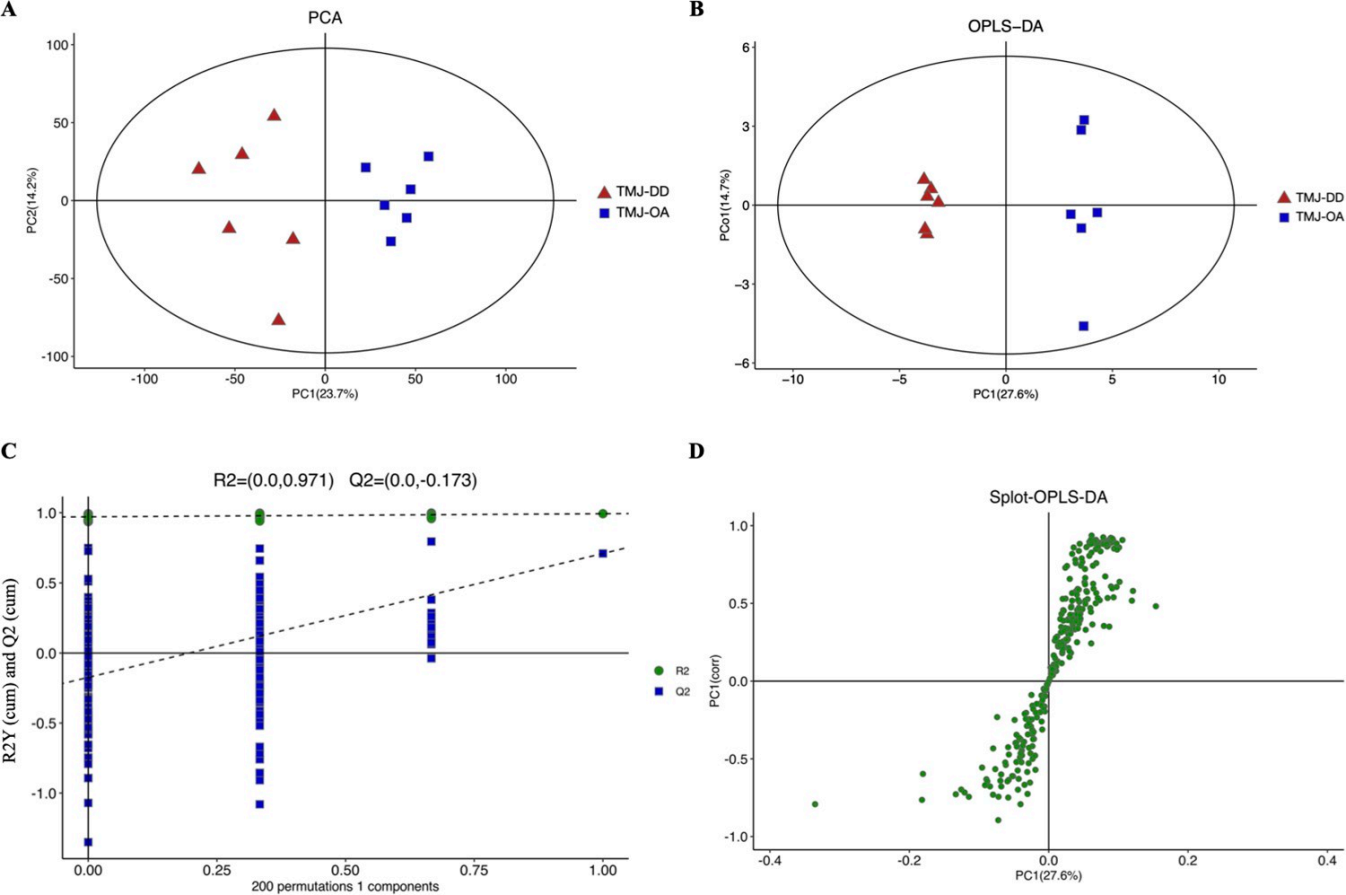


The distribution of DMs profiles among all samples was analyzed and visualized using a PCA method. Notable distinctions were identified in the metabolic profiles of TMJ-OA and TMJ-DD samples (Fig 1A). Subsequently, an OPLS-DA model was utilized to uncover these disparities. The results demonstrated a distinct propensity for separation between TMJ-OA groups and TMJ-DD groups (Fig 1B). In the OPLS-DA model, the values of R2Y and Q2Y were 0.993 and 0.71, showing that the OPLS-DA model was well-suited for recognition analysis. The results of 200 permutation tests showed that the intercepts R2 (0.971) and Q2 (-0.173) supported the conclusion that this model was not over-fitted (Fig 1C).

### Investigating metabolomic profiles in TMJ-OA and TMJ-DD synovial fluid

**Fig 2:**
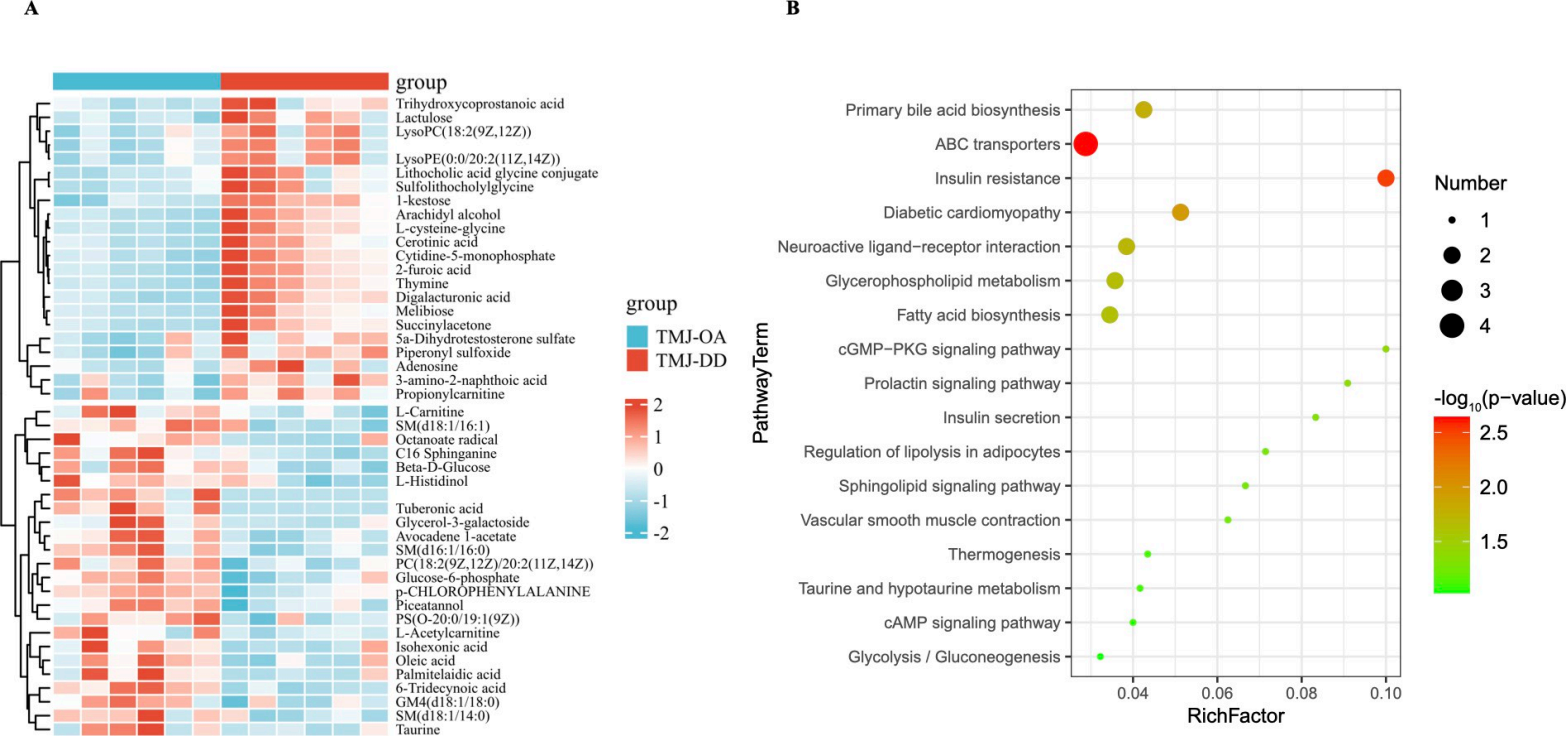


**Fig 3:**
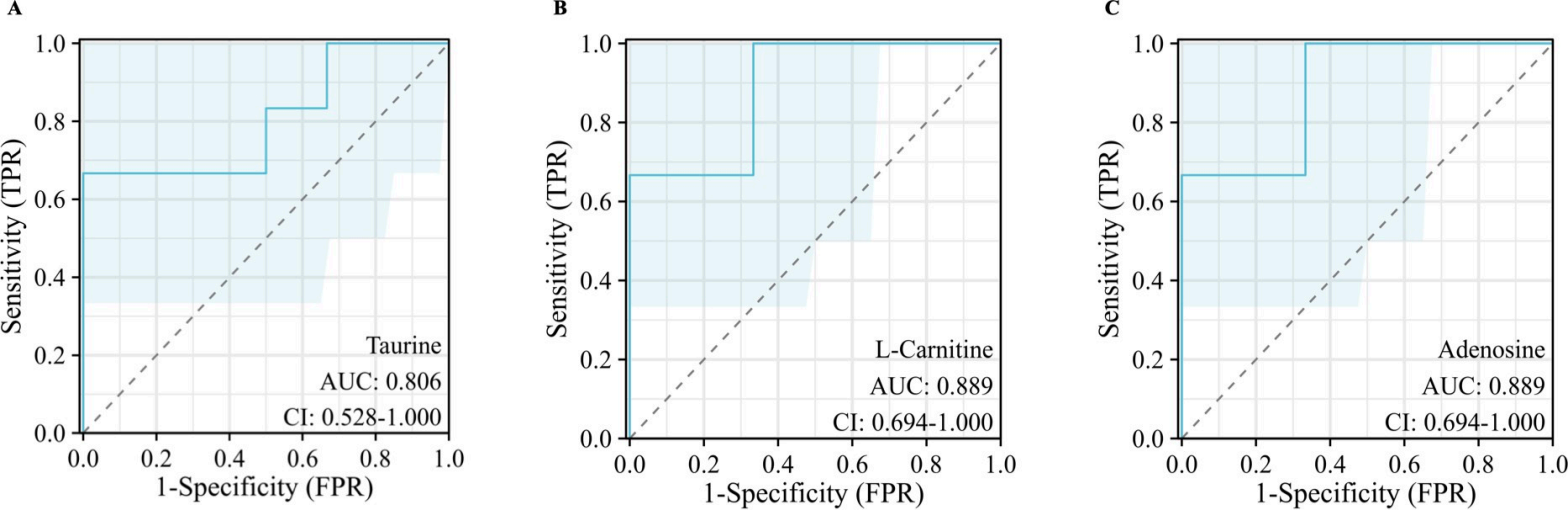


To identify the DMs between TMJ-OA and TMJ-DD, we employed an OPLS-DA model. Consequently, a total of 46 DMs (VIP > 1, p-values < 0.05) were identified as potential DMs between TMJ-OA and TMJ-DD groups (Fig 1D and S1 Table). The 46 DMs were categorized into different chemical classes in Table 1, including fatty acids (27% of the identified metabolites), organic acids (22%), sugars and sugar alcohols (13%), amino acids (4%), amines (7%), and others (27%). The heatmap displayed these 46 DMs (Fig 2A). KEGG analysis revealed that DMs were enriched in the fatty acid biosynthesis, glvcerophospholipid metabolism, sphingolipid signaling pathway, cAMP signaling pathway, glycolysis, gluconeogenesis, and the thermogenesis, taurine and hvpotaurine metabolism (Fig 2B). Through ROC analysis, the results demonstrated that adenosine, L-carnitine, taurine displayed ROC curves with AUC values above 0.8, which were selected as a combination of metabolic biomarkers for clinical diagnosis of TMJ-OA (Fig 3A–3C).

**Table 1:** Classification of metabolites in synovial fluid based on their chemical compositions.

Identified metabolite	
*Amino acids*	
L-cysteine-glycine	L-Histidinol
*Sugars and sugar alcohols*	
Glycerol-3-galactoside	1-kestose
Melibiose	Beta-D-Glucose
Glucose-6-phosphate	Lactulose
*Organic acids*	
Isohexonic acid	Cerotinic acid
2-furoic acid	Digalacturonic acid
Chenodeoxycholic acid 3-amino-2-naphthoic acid	2-linoleoyl-sn-glycero-3-phosphocholine Sulfolithocholylglycine
Chenodeoxycholic acid glycine conjugate	Tuberonic acid
*Fatty acids*	
LysoPC (18:2(9Z,12Z))	LysoPE (0:0/20:2(11Z,14Z))
SM(d18:1/14:0)	GM4(d18:1/18:0)
SM(d16:1/16:0)	SM(d18:1/16:1)
Palmitelaidic acid	Arachidyl alcohol
Oleic acid	6-Tridecynoic acid
C16 Sphinganine	Lithocholic acid glycine conjugate
*Amines*	
Adenosine	Thymine
Cytidine-5-monophosphate	
*Others*	
Octanoate radical	L-acetylcarnitine
L-carnitine	Taurine
Propionylcarnitine	5a-Dihydrotestosterone sulfate
Diethylpropion	Avocadene 1-acetate
Piperonyl sulfoxide	P-chlorophenylalanine

### Identification of DEGs and DE-MTGs

**Fig 4:**
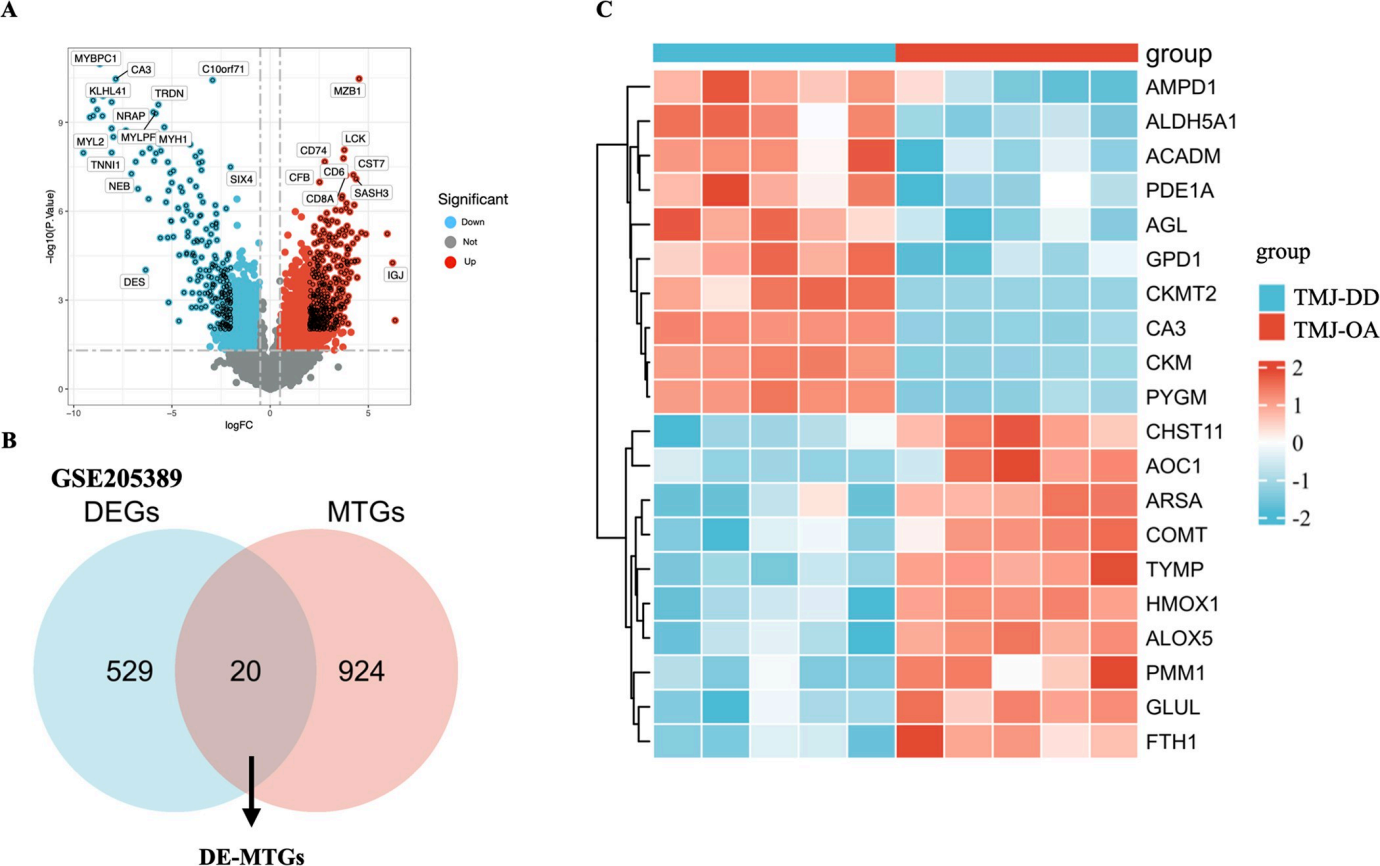


The GSE205389 dataset has essentially the same median, upper and lower quartiles, and maximum and minimum values for the 10 sample (S1A Fig). PCA revealed a large difference between the TMJ-OA and TMJ-DD (S1B Fig), indicating differences in gene expression and good reproducibility during the entire analysis. A total of 549 DEGs (291 upregulated genes and 258 downregulated genes) were identified (S2 Table). The DEGs between the TMJ-OA and TMJ-DD groups are shown in the volcano map (Fig 4A). We identified DE-MTGs by intersecting the MTGs with the DEGs (S3 Table). The 20 DE-MTGs identified by overlapping were selected (Fig 4B). The top 20 DE-MTGs were listed in S4 Table in order of “adj.p.value”. The expression heatmap of the DE-MTGs are displayed in (Fig 4C).

### Enrichment analyses of DE-MTGs

GO and KEGG enrichment analyses were carried out on 20 DE-MTGs. GO analysis showed that upregulated DE-MTGs were mainly enriched in in the amine metabolic process, cellular iron ion homeostasis, dicarboxylic acid catabolic process, while downregulated DE-MTGs were mainly enriched in the glycogen metabolic process, energy reserve metabolic process, and positive regulation of ATP metabolic process, fatty acid beta-oxidation, regulation of glycolytic process (S2A and S2B Fig). KEGG analysis revealed that DE-MTGs were enriched in the ferroptosis, mineral absorption, starch and sucrose metabolism (S2A and S2B Fig).

### Integration analysis of metabolomics and transcriptomics

**Fig 5:**
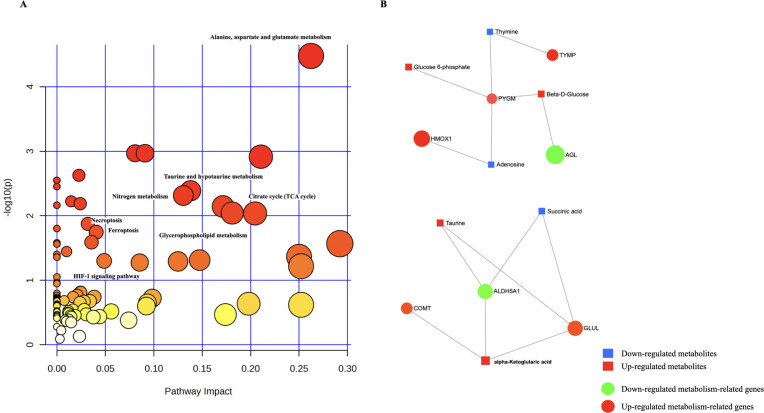


**Fig 6:**
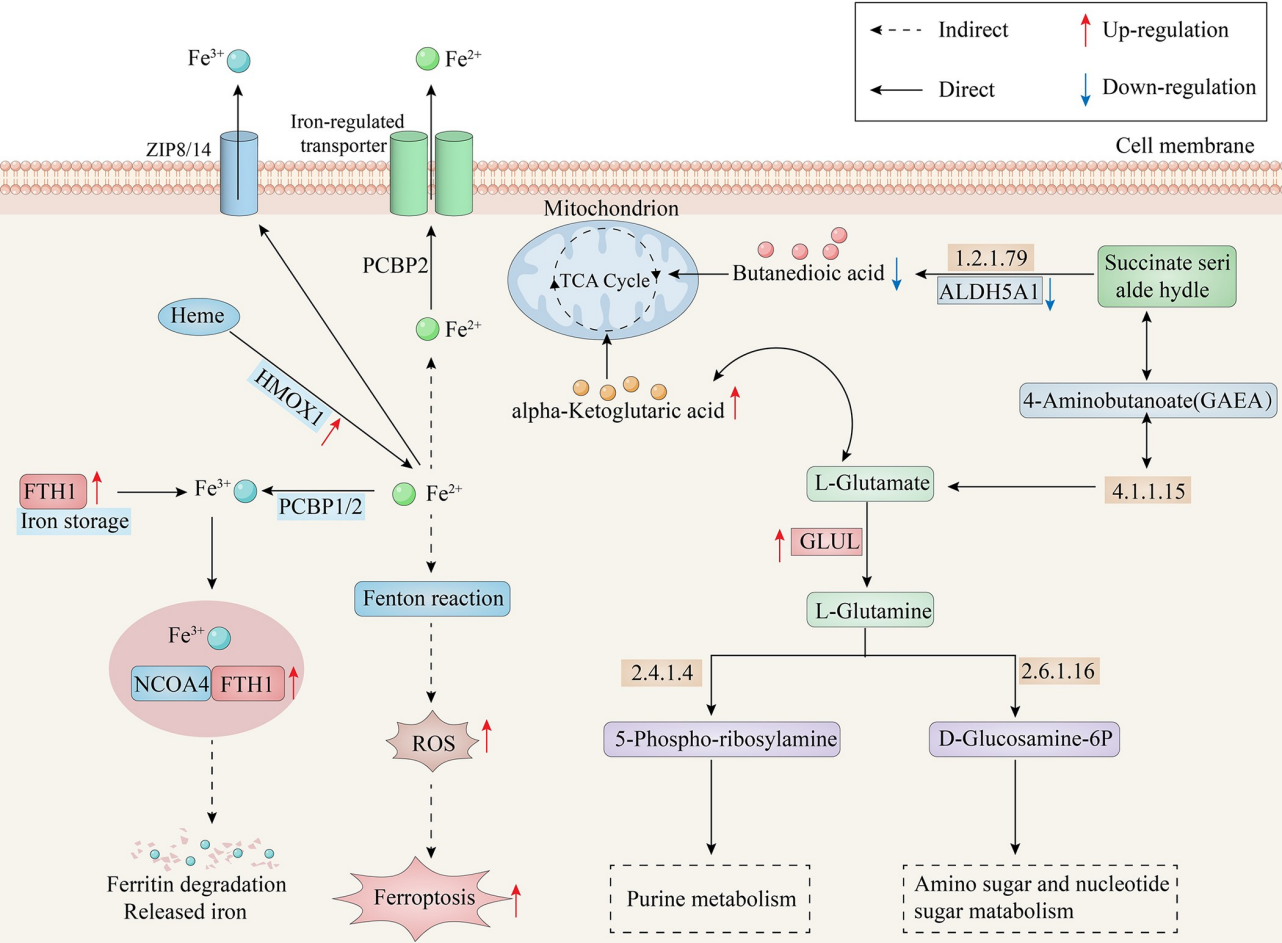


In order to investigate the interactions between DMs and DE-MTGs, we conducted a biological pathway analysis using the OmicsNet module on MetaboAnalyst 5.0. This analysis incorporated 46 DMs and 20 DE-MTGs. According to the integrative analysis, there was significant enrichment observed for the DMs and DE-MTGs mainly in pathways such as tricarboxylic acid (TCA) cycle, alanine, aspartate and glutamate metabolism, ferroptosis pathway, HIF-1 signaling pathway, and additional pathways (Fig 5A and S5 Table). In order to gain a deeper understanding of DE-MTGs and DMs, network analysis was conducted using MetaboAnalyst 5.0 to investigate the relationships between DE-MTGs and DMs (Figs 5B and 6).

## Discussion

TMJ-OA TMJ-OA is characterized by the progressive degeneration of the TMJ tissue. The current diagnosis and treatment of TMJ-OA primarily relies on clinical symptoms, radiological methods and conservative treatments [21]. However, these methods may lack sensitivity in detecting early-stage TMJ-OA in patients and fail to effectively impede the progressive degeneration of articular cartilage. The inadequacy of treatment options to slow down the progression of TMJ-OA may be due to our limited knowledge regarding its underlying mechanisms. Therefore, we are dedicated to exploring the underlying mechanisms and identifying diagnostic biomarkers that play a crucial role in the development of TMJ-OA.

The changes in metabolites have a significant impact on the development and progression of numerous diseases [22]. The application of metabolomics offers distinct advantages in diagnosing, evaluating treatments, and exploring the underlying mechanisms of OA [23, 24]. By employing metabolomic analysis, potential pathogenic mechanisms and diagnostic biomarkers for TMJ-OA can be elucidated. Owing to its inherent sensitivity, metabolomics presents a highly effective approach for discovering biomarkers and enables a deeper understanding of the dynamic nature of metabolic pathways and their influence on biological processes.

The availability of easily obtainable biological fluids, such as synovial fluid, serum, and urine, offers practical opportunities for conducting metabolic studies and distinct merits in assessing the physiological state of organisms, offering valuable opportunities for comprehensive monitoring and evaluation of their health [25, 26]. In comparison to synovial fluid collection, blood sampling is a less invasive method. However, it is crucial to consider that metabolites from the affected joint need to pass through the synovium before being detected in the bloodstream. This transit may lead to the dilution of potential biomarkers and make them vulnerable to degradation within the circulatory system. Due to its direct interaction with the diseased joint tissue, synovial fluid exhibits great potential for identifying biomarkers in OA [27]. In recent years, several studies have successfully identified and distinguished the metabolic profiles in the synovial fluid of OA patients [25, 26]. According to a study, the composition and concentrations of metabolites were found to be changed in osteoarthritic synovial fluid [21].

This study represents the first investigation into alterations in metabolite profiles in synovial fluid from TMJ-OA. From the synovial fluid of TMJ-OA patients, we identified 46 metabolites, which were divided into different chemical classes such as amino acids, sugars and sugar alcohols, organic acids, fatty acids, amines, and otehrs. Notably, OPLS-DA analysis revealed clear differentiation between the metabolite profiles of these two groups. These 46 metabolites were found to be associated with fatty acid biosynthesis, glvcerophospholipid metabolism, sphingolipid signaling pathway, cAMP signaling pathway, glycolysis, gluconeogenesis, and the thermogenesis, taurine and hvpotaurine metabolism. The pathway analysis demonstrated changes in fatty acid biosynthesis. Importantly, fatty acids including oleic acid, palmitelaidic acid, and 6-tridecynoic acid exhibited higher levels in the TMJ-OA groups. Earlier investigations have reported an increased accumulation of cholesterol and fatty acids in cartilage with the advancement of OA severity [28, 29]. Glycerophospholipids and sphingolipids, including LysoPC(18:2(9Z,12Z)), LysoPE(0:0/20:2(11Z,14Z)), SM(d18:1/14:0), SM(d18:1/16:1), SM(d16:1/16:0), exhibited significant upregulation in synovial fluid of TM-OA. Widely distributed in cell membranes, glycosphingolipids play essential roles in tissue development and function [30]. The inhibition of glycosphingolipid synthesis led to a reduced activation of osteoclasts, indicating an intensified catabolism of sclerotic subchondral bone [31]. In addition, sphingolipids have the capacity to modify synovial inflammation and impact the repair mechanisms of damaged joints [32]. Briefly, fatty acid biosynthesis, glycerophospholipids and sphingolipids are crucial in regulating the pathological progression of TMJ-OA.

As the principal energy supplier, adenosine triphosphate (ATP) underpins the foundation of human metabolism, enabling a plethora of vital metabolic reactions and physiological functions, which is associated with cAMP signaling pathway, glycolysis, gluconeogenesis metabolism, and the thermogenesis. Through purinergic signaling, ATP plays a vital regulatory role in chondrocyte function and contributes significantly to the maintenance and repair of cartilage [33, 34]. The levels of adenosine, L-carnitine, cytidine-5-monophosphate, and succinylacetone, which are associated with ATP production and metabolism, were found to be significantly altered in patients with TMJ-OA in this study. The hypoxia and mitochondrial dysfunction within the microenvironment of chronic inflammation lead to reduced ATP synthesis, resulting in decreased adenosine levels [35]. Due to the avascular nature of mature articular cartilage, chondrocytes primarily rely on anaerobic metabolism to support, preserve, and restore the extracellular matrix (ECM) of cartilage, leading to a limited production of ATP [36]. In chondrocytes afflicted by osteoarthritis, there is an augmented rate of anaerobic glycolysis, thereby generating ample ATP for the reparative process [35]. Extracellular adenosine, derived from the conversion of ATP released by chondrocytes, plays a critical role in preventing the phenotypic changes in chondrocytes associated with the development of OA. Inflammation, injury, or aging can disrupt this mechanism, causing a decrease ATP levels along with extracellular adenosine and phenotypic changes in chondrocytes, characterized by an increased expression of matrix metalloproteinase (MMPs) or collagens associated with cartilage hypertrophy [37]. L-carnitine promotes the entry of oxygen into the TCA cycle, facilitating ATP synthesis and ultimately leading to a decline in oxygen concentration and the suppression of reactive oxygen species (ROS) formation [38]. These findings suggest that glycolysis, gluconeogenesis and thermogenesis pathway associated with the ATP production exerts predominant control over energy generation in the TMJ-OA.

In addition, our study identified L-carnitine, taurine, and adenosine as potential biomarkers capable of differentiating individuals with TMJ-OA from healthy controls. The results of an in vitro study on human primary chondrocytes revealed that L-carnitine exhibited high effectiveness in promoting cell proliferation and inducing ATP synthesis [39]. The targeted metabolomics investigation revealed increased taurine levels in osteophyte cartilage samples compared to control samples, suggesting its potential role in collagen degradation [40]. Adenosine plays a significant role in supporting the structural integrity and optimal function of cartilage [41]. Stimulation of the A2AR by adenosine leads to an increase in intracellular cAMP levels within chondrocytes, while simultaneously suppressing the production of nitric oxide [42].

It’s generally accepted that molecular and metabolic factors initiate the onset of OA, crucially characterized by a metabolic shift from oxidative phosphorylation to glycolysis, which impacts energy production and predominantly affects proinflammatory pathways [43]. Chondrocytes in healthy joints have the capacity to stimulate mitochondrial respiration and curtail the synthesis rate of reactive nitrogen and oxygen, thus satisfying energy demands under severe nutritional stress and enhancing survival [44]. In the early stages of OA, articular cartilage displays metabolic adaptability and adjustability in response to stressful environmental conditions, contrasting the less adaptable nature observed in the later stages [45]. However, the metabolism-related genes, which could possibly stimulate metabolic changes, have not yet completely understood.

For the identification of DE-MTGs, we identified 20 DE-MTGs, including 10 upregulated genes (TYMP, HMOX1, ALOX5, GLUL, CHST11, ARSA, FTH1, COMT, PMM1, AOC1) and 10 downregulated genes (ACADM, ALDH5A1, AMPD1, CA3, CKMT2, COMT, GPD1, PDE1A, PYGM, AGL). The integrative analysis revealed that the ALDH5A1, GLUL and alpha-Ketoglutaric acid (α-KG), Butanedioic acid were highly enriched in alanine, aspartate and glutamate metabolism pathways, FTH1 and HMOX1 genes were highly enriched in ferroptosis, and alpha-Ketoglutaric acid, Butanedioic acid were highly enriched in TCA cycle. The TCA cycle, functioning as the ultimate converging oxidative pathway for carbohydrates, fats, and amino acids, plays a paramount role in supplying cells with the primary source of ATP derived from metabolic fuels [46]. Blocking ATP synthesis and inhibiting pyruvate dehydrogenase in the TCA cycle leads to a delay in chondrocyte maturation, emphasizing the significance of acetyl-CoA as a regulator of maturation [47]. Metabolic changes in amino acid pathways also play a role in OA pathology. The arginine metabolism pathway, specifically the iNOS-mediated conversion of arginine into NO in chondrocytes, is closely linked to the pathogenesis of OA [48]. Chondrocytes have been found to exhibit glutamate metabolism, involving the release of glutamine and the expression of glutamate transporter N-methyl-D-aspartate (NMDA) glutamate receptors (NMDAR) [49]. Inhibition of NMDAR restores PER2 and BMAL1 expression in chondrocytes and leads to a reduction in MMP-13 and Collagen X (COL10A1) levels, indicating its potential role in the development of OA [50]. Chondrocyte ferroptosis, a newly recognized mode of programmed cell death driven by iron-dependent lipid peroxidation, has been identified as a contributor to OA progression [51]. Comparative analysis has demonstrated that the levels of iron in the synovial fluids of OA patients are notably elevated in comparison to control groups [52]. Research has demonstrated that inducing chondrocyte ferroptosis leads to elevated MMP13 expression and decreased COL2A1 expression [53]. In conclusion, patients with TMJ-OA may exhibit metabolic disorders in their synovial tissue cells.

α-Ketoglutarate (α-KG) plays a significant role in a variety of metabolic processes, including the TCA cycle and production of amino acids [54, 55]. α-KG has been shown in previous research to promote the proliferation of chondrocytes and exerts a relieving effect on OA by regulating cartilage metabolism and inhibiting inflammation [56, 57]. By enhancing basal respiration, ATP-linked mitochondrial respiration, α-KG markedly ameliorates the impaired mitochondrial respiration induced by IL-1β [58]. This study revealed that the two upregulated genes FTH1 and HMOX1 were highly enriched in ferroptosis. Single-cell transcriptomic analysis of osteoarthritic cartilage has revealed a significant enrichment of ferroptosis and an increased expression of genes associated with ferroptosis, including FTH1 and HMOX1 [59]. The precise maintenance of cellular iron balance relies on the intricate regulatory role of FTH1, a prominent protein involved in cellular iron storage [60]. It has been consistently observed in previous investigations that there exists a positive relationship between higher FTH1 mRNA expression and a higher prevalence of hand osteoarthritis [59]. HMOX1, an essential enzyme involved in heme catabolism, plays a key role in ferroptosis and significantly alleviates erastin-induced ferroptosis in renal epithelial cells [61]. The induction of ferroptosis can be attributed to the upregulation of HMOX1, which leads to the accumulation of ROS and intracellular ferric iron levels via the inhibition of GPX4 expression [62].

## Conclusions

The present study has revealed several early metabolic signatures for TMJ-OA, which might advance prediction and prevention of TMJ-OA in Chinese population. A total of 46 metabolites exhibited significant alterations in the synovial fluid of TMJ-OA patients compared to control groups. L-carnitine, taurine, and adenosine are potential biomarkers that may be helpful in diagnosing TMJ-OA. Furthermore, an integrated analysis of metabolomics and transcriptomics suggests that the progression of TMJ-OA are associated with disruptions in metabolic pathways, such as TCA cycle, alanine, aspartate and glutamate metabolism, ferroptosis. However, it is important to acknowledge that the current study has some limitations, such as a small sample size and the absence of verification analysis. Firstly, in the current study, TMJ-OA was diagnosed based on the clinical criteria without laboratory confirmation. Further studies to link peripheral metabolic changes to pathophysiology markers, genetic findings and bone formation profiles are recommended. And the candidate metabolites from this study should be validated in an independent and larger replication sample of Chinese adults. Secondly, all gene-associated results were obtained through analysis of the GEO database and lacked independent verification using our own dataset. Therefore, conducting a follow-up study with a larger sample size is crucial to validate all the findings presented in this study.

## Supporting information

S1 Fig **(A)** Boxplot of gene probe expression levels among samples. There was no significant difference in the median and the upper and lower quartile. **(B)** PCA principal-component analysis.
(DOCX)

S2 Fig Functional enrichment of the DE-MTGs. **(A)** GO/KEGG analysis of upregulated DE-MTGs. **(B)** GO/KEGG analysis of downregulated DE-MTGs.
(DOCX)

S1 Table (DOCX)

S2 Table (XLSX)

S3 Table (XLSX)

S4 Table (DOCX)

S5 Table (DOCX)

## References

[1] Badel T, Zadravec D, Basic Kes V, Smoljan M, Kocijan Lovko S, Zavoreo I, et al. Orofacial Pain—Diagnostic and Therapeutic Challenges. Acta Clin Croat. 2019;58(Suppl 1):82–9. doi: 10.20471/acc.2019.58.s1.12 31741564 PMC6813472

[2] Xu J, Liu Y, Deng M, Li J, Cai H, Meng Q, et al. MicroRNA221-3p modulates Ets-1 expression in synovial fibroblasts from patients with osteoarthritis of temporomandibular joint. Osteoarthritis Cartilage. 2016;24(11):2003–11. doi: 10.1016/j.joca.2016.06.011 27349463

[3] Yamashita-Futani Y, Jokaji R, Ooi K, Kobayashi K, Kanakis I, Liu K, et al. Metalloelastase-12 is involved in the temporomandibular joint inflammatory response as well as cartilage degradation by aggrecanases in STR/Ort mice. Biomed Rep. 2021;14(6):51. doi: 10.3892/br.2021.1427 33859822 PMC8042671

[4] Machon V, Hirjak D, Lukas J. Therapy of the osteoarthritis of the temporomandibular joint. J Craniomaxillofac Surg. 2011;39(2):127–30. doi: 10.1016/j.jcms.2010.04.010 20692843

[5] de Souza RF, Lovato da Silva CH, Nasser M, Fedorowicz Z, Al-Muharraqi MA. Interventions for the management of temporomandibular joint osteoarthritis. Cochrane Database Syst Rev. 2012;2012(4):CD007261. doi: 10.1002/14651858.CD007261.pub2 22513948 PMC6513203

[6] Atherton HJ, Jones OA, Malik S, Miska EA, Griffin JL. A comparative metabolomic study of NHR-49 in Caenorhabditis elegans and PPAR-alpha in the mouse. FEBS Lett. 2008;582(12):1661–6. doi: 10.1016/j.febslet.2008.04.020 18435929 PMC6997030

[7] Zhou J, Chen J, Hu C, Xie Z, Li H, Wei S, et al. Exploration of the serum metabolite signature in patients with rheumatoid arthritis using gas chromatography-mass spectrometry. J Pharm Biomed Anal. 2016;127:60–7. doi: 10.1016/j.jpba.2016.02.004 26879423

[8] Adams SB Jr., Setton LA, Nettles DL. The role of metabolomics in osteoarthritis research. J Am Acad Orthop Surg. 2013;21(1):63–4. doi: 10.5435/JAAOS-21-01-63 23281473 PMC3660045

[9] Gowda GA, Djukovic D. Overview of mass spectrometry-based metabolomics: opportunities and challenges. Methods Mol Biol. 2014;1198:3–12. doi: 10.1007/978-1-4939-1258-2_1 25270919 PMC4336784

[10] Mobasheri A, Bondy CA, Moley K, Mendes AF, Rosa SC, Richardson SM, et al. Facilitative glucose transporters in articular chondrocytes. Expression, distribution and functional regulation of GLUT isoforms by hypoxia, hypoxia mimetics, growth factors and pro-inflammatory cytokines. Adv Anat Embryol Cell Biol. 2008;200:1 p following vi, -84. 18841755

[11] Rahmati M, Nalesso G, Mobasheri A, Mozafari M. Aging and osteoarthritis: Central role of the extracellular matrix. Ageing Res Rev. 2017;40:20–30. doi: 10.1016/j.arr.2017.07.004 28774716

[12] Damyanovich AZ, Staples JR, Chan AD, Marshall KW. Comparative study of normal and osteoarthritic canine synovial fluid using 500 MHz 1H magnetic resonance spectroscopy. J Orthop Res. 1999;17(2):223–31. doi: 10.1002/jor.1100170211 10221839

[13] Lacitignola L, Fanizzi FP, Francioso E, Crovace A. 1H NMR investigation of normal and osteo-arthritic synovial fluid in the horse. Vet Comp Orthop Traumatol. 2008;21(1):85–8. doi: 10.3415/VCOT-06-12-0101 18288348

[14] Rousseau J, Garnero P. Biological markers in osteoarthritis. Bone. 2012;51(2):265–77. doi: 10.1016/j.bone.2012.04.001 22538364

[15] Schiffman E, Ohrbach R, Truelove E, Look J, Anderson G, Goulet JP, et al. Diagnostic Criteria for Temporomandibular Disorders (DC/TMD) for Clinical and Research Applications: recommendations of the International RDC/TMD Consortium Network* and Orofacial Pain Special Interest Groupdagger. J Oral Facial Pain Headache. 2014;28(1):6–27. doi: 10.11607/jop.1151 24482784 PMC4478082

[16] Fan PD, Xiong X, Cheng QY, Xiang J, Zhou XM, Yi YT, et al. Risk estimation of degenerative joint disease in temporomandibular disorder patients with different types of sagittal and coronal disc displacements: MRI and CBCT analysis. J Oral Rehabil. 2023;50(1):12–23. doi: 10.1111/joor.13385 36282624

[17] Ma FY, Zhang XM, Li Y, Zhang M, Tu XH, Du LQ. Identification of phenolics from miracle berry (Synsepalum dulcificum) leaf extract and its antiangiogenesis and anticancer activities. Front Nutr. 2022;9:970019. Epub 2022/09/02. doi: 10.3389/fnut.2022.970019 36046137 PMC9420939

[18] Lu X, Huang L, Chen Y, Hu L, Zhong R, Chen L, et al. Effect of DHA-Enriched Phospholipids from Fish Roe on Rat Fecal Metabolites: Untargeted Metabolomic Analysis. Foods. 2023;12(8). Epub 2023/04/28. doi: 10.3390/foods12081687 37107484 PMC10137559

[19] Ritchie ME, Phipson B, Wu D, Hu Y, Law CW, Shi W, et al. limma powers differential expression analyses for RNA-sequencing and microarray studies. Nucleic Acids Res. 2015;43(7):e47. doi: 10.1093/nar/gkv007 25605792 PMC4402510

[20] Yu G, Wang LG, Han Y, He QY. clusterProfiler: an R package for comparing biological themes among gene clusters. OMICS. 2012;16(5):284–7. doi: 10.1089/omi.2011.0118 22455463 PMC3339379

[21] Cui C, Zheng L, Fan Y, Zhang J, Xu R, Xie J, et al. Parathyroid hormone ameliorates temporomandibular joint osteoarthritic-like changes related to age. Cell Prolif. 2020;53(4):e12755. doi: 10.1111/cpr.12755 32154622 PMC7162802

[22] Li Q, Gu W, Ma X, Liu Y, Jiang L, Feng R, et al. Amino Acid and Biogenic Amine Profile Deviations in an Oral Glucose Tolerance Test: A Comparison between Healthy and Hyperlipidaemia Individuals Based on Targeted Metabolomics. Nutrients. 2016;8(6). doi: 10.3390/nu8060379 27338465 PMC4924220

[23] Mickiewicz B, Heard BJ, Chau JK, Chung M, Hart DA, Shrive NG, et al. Metabolic profiling of synovial fluid in a unilateral ovine model of anterior cruciate ligament reconstruction of the knee suggests biomarkers for early osteoarthritis. J Orthop Res. 2015;33(1):71–7. doi: 10.1002/jor.22743 25283885

[24] Holmes E, Wilson ID, Nicholson JK. Metabolic phenotyping in health and disease. Cell. 2008;134(5):714–7. doi: 10.1016/j.cell.2008.08.026 18775301

[25] Nguyen LT, Sharma AR, Chakraborty C, Saibaba B, Ahn ME, Lee SS. Review of Prospects of Biological Fluid Biomarkers in Osteoarthritis. Int J Mol Sci. 2017;18(3). doi: 10.3390/ijms18030601 28287489 PMC5372617

[26] de Sousa EB, Dos Santos GCJ, Duarte MEL, Moura VN, Aguiar DP. Metabolomics as a promising tool for early osteoarthritis diagnosis. Braz J Med Biol Res. 2017;50(11):e6485. doi: 10.1590/1414-431X20176485 28953990 PMC5609603

[27] Huffman KM, Bowers JR, Dailiana Z, Huebner JL, Urbaniak JR, Kraus VB. Synovial fluid metabolites in osteonecrosis. Rheumatology (Oxford). 2007;46(3):523–8. doi: 10.1093/rheumatology/kel302 17003168

[28] Cillero-Pastor B, Eijkel G, Kiss A, Blanco FJ, Heeren RM. Time-of-flight secondary ion mass spectrometry-based molecular distribution distinguishing healthy and osteoarthritic human cartilage. Anal Chem. 2012;84(21):8909–16. doi: 10.1021/ac301853q 22950553

[29] Lippiello L, Walsh T, Fienhold M. The association of lipid abnormalities with tissue pathology in human osteoarthritic articular cartilage. Metabolism. 1991;40(6):571–6. doi: 10.1016/0026-0495(91)90046-y 1865821

[30] Seito N, Yamashita T, Tsukuda Y, Matsui Y, Urita A, Onodera T, et al. Interruption of glycosphingolipid synthesis enhances osteoarthritis development in mice. Arthritis Rheum. 2012;64(8):2579–88. doi: 10.1002/art.34463 22391889 PMC3382033

[31] Ersek A, Xu K, Antonopoulos A, Butters TD, Santo AE, Vattakuzhi Y, et al. Glycosphingolipid synthesis inhibition limits osteoclast activation and myeloma bone disease. J Clin Invest. 2015;125(6):2279–92. doi: 10.1172/JCI59987 25915583 PMC4518690

[32] Kosinska MK, Liebisch G, Lochnit G, Wilhelm J, Klein H, Kaesser U, et al. Sphingolipids in human synovial fluid—a lipidomic study. PLoS One. 2014;9(3):e91769. doi: 10.1371/journal.pone.0091769 24646942 PMC3960152

[33] Croucher LJ, Crawford A, Hatton PV, Russell RG, Buttle DJ. Extracellular ATP and UTP stimulate cartilage proteoglycan and collagen accumulation in bovine articular chondrocyte pellet cultures. Biochim Biophys Acta. 2000;1502(2):297–306. doi: 10.1016/s0925-4439(00)00055-7 11040454

[34] Millward-Sadler SJ, Wright MO, Flatman PW, Salter DM. ATP in the mechanotransduction pathway of normal human chondrocytes. Biorheology. 2004;41(3–4):567–75. 15299287

[35] Magni G, Ceruti S. Adenosine Signaling in Autoimmune Disorders. Pharmaceuticals (Basel). 2020;13(9). doi: 10.3390/ph13090260 32971792 PMC7558305

[36] Sophia Fox AJ, Bedi A, Rodeo SA. The basic science of articular cartilage: structure, composition, and function. Sports Health. 2009;1(6):461–8. doi: 10.1177/1941738109350438 23015907 PMC3445147

[37] Corciulo C, Lendhey M, Wilder T, Schoen H, Cornelissen AS, Chang G, et al. Endogenous adenosine maintains cartilage homeostasis and exogenous adenosine inhibits osteoarthritis progression. Nat Commun. 2017;8:15019. doi: 10.1038/ncomms15019 28492224 PMC5437286

[38] Lee BJ, Lin JS, Lin YC, Lin PT. Effects of L-carnitine supplementation on oxidative stress and antioxidant enzymes activities in patients with coronary artery disease: a randomized, placebo-controlled trial. Nutr J. 2014;13:79. doi: 10.1186/1475-2891-13-79 25092108 PMC4125592

[39] Stoppoloni D, Politi L, Dalla Vedova P, Messano M, Koverech A, Scandurra R, et al. L-carnitine enhances extracellular matrix synthesis in human primary chondrocytes. Rheumatol Int. 2013;33(9):2399–403. doi: 10.1007/s00296-012-2373-9 22451022

[40] Yang G, Zhang H, Chen T, Zhu W, Ding S, Xu K, et al. Metabolic analysis of osteoarthritis subchondral bone based on UPLC/Q-TOF-MS. Anal Bioanal Chem. 2016;408(16):4275–86. doi: 10.1007/s00216-016-9524-x 27074781

[41] Bekisz JM, Lopez CD, Corciulo C, Mediero A, Coelho PG, Witek L, et al. The Role of Adenosine Receptor Activation in Attenuating Cartilaginous Inflammation. Inflammation. 2018;41(4):1135–41. doi: 10.1007/s10753-018-0781-z 29656316

[42] Tesch AM, MacDonald MH, Kollias-Baker C, Benton HP. Endogenously produced adenosine regulates articular cartilage matrix homeostasis: enzymatic depletion of adenosine stimulates matrix degradation. Osteoarthritis Cartilage. 2004;12(5):349–59. doi: 10.1016/j.joca.2004.01.002 15094133

[43] Zhang L, Hu J, Athanasiou KA. The role of tissue engineering in articular cartilage repair and regeneration. Crit Rev Biomed Eng. 2009;37(1–2):1–57. doi: 10.1615/critrevbiomedeng.v37.i1-2.10 20201770 PMC3146065

[44] Lane RS, Fu Y, Matsuzaki S, Kinter M, Humphries KM, Griffin TM. Mitochondrial respiration and redox coupling in articular chondrocytes. Arthritis Res Ther. 2015;17(1):54. doi: 10.1186/s13075-015-0566-9 25889867 PMC4384316

[45] Zhang M, Theleman JL, Lygrisse KA, Wang J. Epigenetic Mechanisms Underlying the Aging of Articular Cartilage and Osteoarthritis. Gerontology. 2019;65(4):387–96. doi: 10.1159/000496688 30970348 PMC9150844

[46] Akram M. Citric acid cycle and role of its intermediates in metabolism. Cell Biochem Biophys. 2014;68(3):475–8. doi: 10.1007/s12013-013-9750-1 24068518

[47] Kosai A, Horike N, Takei Y, Yamashita A, Fujita K, Kamatani T, et al. Changes in acetyl-CoA mediate Sik3-induced maturation of chondrocytes in endochondral bone formation. Biochem Biophys Res Commun. 2019;516(4):1097–102. doi: 10.1016/j.bbrc.2019.06.139 31280862

[48] Wu G. Amino acids: metabolism, functions, and nutrition. Amino Acids. 2009;37(1):1–17. doi: 10.1007/s00726-009-0269-0 19301095

[49] Piepoli T, Mennuni L, Zerbi S, Lanza M, Rovati LC, Caselli G. Glutamate signaling in chondrocytes and the potential involvement of NMDA receptors in cell proliferation and inflammatory gene expression. Osteoarthritis Cartilage. 2009;17(8):1076–83. doi: 10.1016/j.joca.2009.02.002 19233337

[50] Kalev-Zylinska ML, Hearn JI, Rong J, Zhu M, Munro J, Cornish J, et al. Altered N-methyl D-aspartate receptor subunit expression causes changes to the circadian clock and cell phenotype in osteoarthritic chondrocytes. Osteoarthritis Cartilage. 2018;26(11):1518–30. doi: 10.1016/j.joca.2018.06.015 30031924

[51] Wang S, Li W, Zhang P, Wang Z, Ma X, Liu C, et al. Mechanical overloading induces GPX4-regulated chondrocyte ferroptosis in osteoarthritis via Piezo1 channel facilitated calcium influx. J Adv Res. 2022;41:63–75. doi: 10.1016/j.jare.2022.01.004 36328754 PMC9637484

[52] Yao X, Sun K, Yu S, Luo J, Guo J, Lin J, et al. Chondrocyte ferroptosis contribute to the progression of osteoarthritis. J Orthop Translat. 2021;27:33–43. doi: 10.1016/j.jot.2020.09.006 33376672 PMC7750492

[53] Yazar M, Sarban S, Kocyigit A, Isikan UE. Synovial fluid and plasma selenium, copper, zinc, and iron concentrations in patients with rheumatoid arthritis and osteoarthritis. Biol Trace Elem Res. 2005;106(2):123–32. doi: 10.1385/BTER:106:2:123 16116244

[54] Bott M. Offering surprises: TCA cycle regulation in Corynebacterium glutamicum. Trends Microbiol. 2007;15(9):417–25. doi: 10.1016/j.tim.2007.08.004 17764950

[55] Zhang B, Peng H, Zhou M, Bao L, Wang C, Cai F, et al. Targeting BCAT1 Combined with alpha-Ketoglutarate Triggers Metabolic Synthetic Lethality in Glioblastoma. Cancer Res. 2022;82(13):2388–402. doi: 10.1158/0008-5472.CAN-21-3868 35499760 PMC9256772

[56] Singh D, Vishnoi T, Kumar A. Effect of alpha-ketoglutarate on growth and metabolism of cells cultured on three-dimensional cryogel matrix. Int J Biol Sci. 2013;9(5):521–30. doi: 10.7150/ijbs.4962 23781146 PMC3677688

[57] Liu L, Zhang W, Liu T, Tan Y, Chen C, Zhao J, et al. The physiological metabolite alpha-ketoglutarate ameliorates osteoarthritis by regulating mitophagy and oxidative stress. Redox Biol. 2023;62:102663. doi: 10.1016/j.redox.2023.102663 36924682 PMC10026041

[58] D’Amico D, Olmer M, Fouassier AM, Valdes P, Andreux PA, Rinsch C, et al. Urolithin A improves mitochondrial health, reduces cartilage degeneration, and alleviates pain in osteoarthritis. Aging Cell. 2022;21(8):e13662. doi: 10.1111/acel.13662 35778837 PMC9381911

[59] Li H, Jiang X, Xiao Y, Zhang Y, Zhang W, Doherty M, et al. Combining single-cell RNA sequencing and population-based studies reveals hand osteoarthritis-associated chondrocyte subpopulations and pathways. Bone Res. 2023;11(1):58. doi: 10.1038/s41413-023-00292-7 37914703 PMC10620170

[60] Arosio P, Ingrassia R, Cavadini P. Ferritins: a family of molecules for iron storage, antioxidation and more. Biochim Biophys Acta. 2009;1790(7):589–99. doi: 10.1016/j.bbagen.2008.09.004 18929623

[61] Adedoyin O, Boddu R, Traylor A, Lever JM, Bolisetty S, George JF, et al. Heme oxygenase-1 mitigates ferroptosis in renal proximal tubule cells. Am J Physiol Renal Physiol. 2018;314(5):F702–F14. doi: 10.1152/ajprenal.00044.2017 28515173 PMC6031916

[62] Lin H, Chen X, Zhang C, Yang T, Deng Z, Song Y, et al. EF24 induces ferroptosis in osteosarcoma cells through HMOX1. Biomed Pharmacother. 2021;136:111202. doi: 10.1016/j.biopha.2020.111202 33453607

