# Correction: Integration of metabolomics and transcriptomics provides insights into the molecular mechanism of temporomandibular joint osteoarthritis

**DOI:** 10.1371/journal.pone.0319428

**Published:** 2025-02-11

**Authors:** Palati Tuerxun, Takkun Ng, Ke Zhao, Ping Zhu

In Fig 2, there are errors in the names of chemical metabolites in panel A. The chemical metabolites “Piceatannol” and “Trihydroxycoprostanoic acid” should be “2-linoleoyl-sn-glycero-3-phosphocholine” and “Diethylpropion (metabolite XI Glucuronide)” and the “Alpha-ketoglutarate” and “Butanedioic acid” metabolites are omitted. Also, the caption is missing. Please see the correct Fig 2 and its complete caption here.

**Fig 1:**
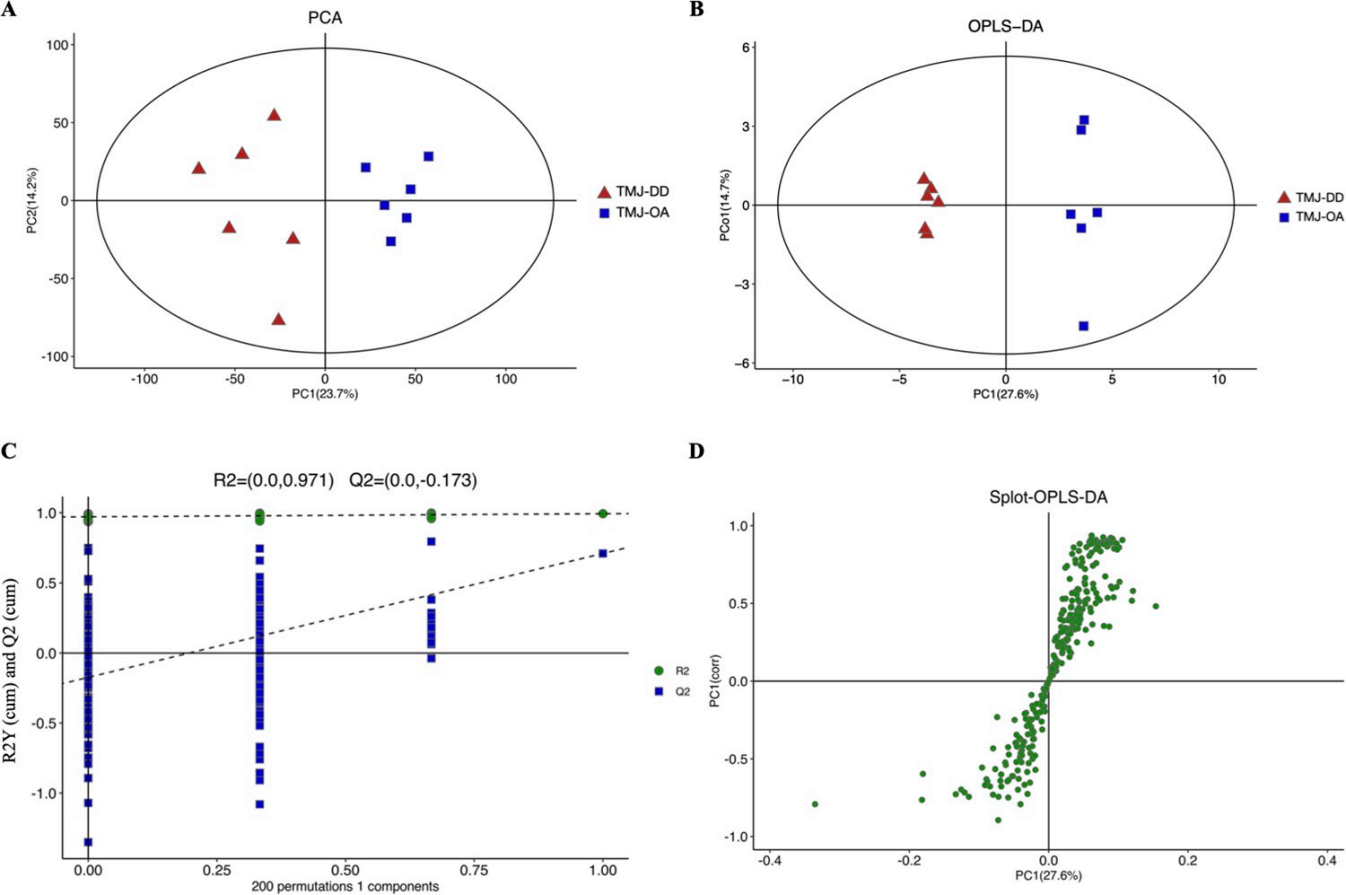
Comprehensive analysis of the synovial fluid metabolomic profiles of TMJ-OA and TMJ-DD samples using PCA and OPLS-DA analysis. **(A)** The PCA plot highlights the distinct distribution of the TMJ-OA and the TMJ-DD groups, indicating dissimilarities in the metabolite profiles of synovial fluid between these two groups. **(B)** The OPLS-DA analysis. **(C)** Evaluation of OPLS-DA model through permutation tests [intercept, R2 = 0.971; Q2 = −0.173]. **(D)** The Splot was constructed by comparing synovial fluid samples between the TMJ-OA and TMJ-DD groups.

**Fig 2:**
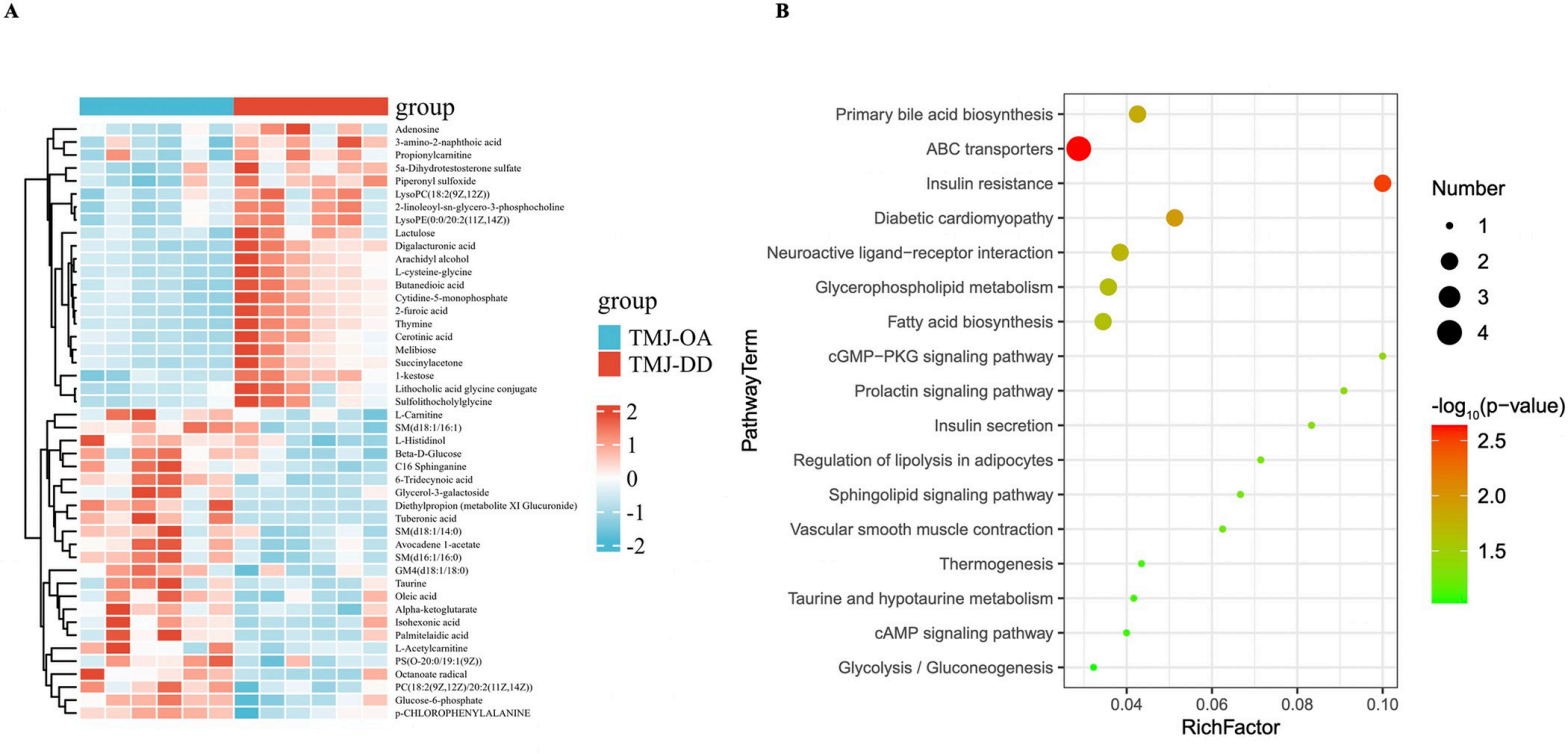
Investigating metabolomic profiles of TMJ-OA and TMJ-DD samples. (A) Heatmap illustrating the 46 DMs between TMJ-OA and TMJ-DD groups. (B) Visualization of pathway analysis results for 46 DMs with significant differences between TMJ-OA and TMJ-DD groups.

In Fig 5, the color of the circle that corresponds to the PYGM gene are switched and its caption is missing. Please see the correct Fig 5 and its complete caption here.

**Fig 3:**
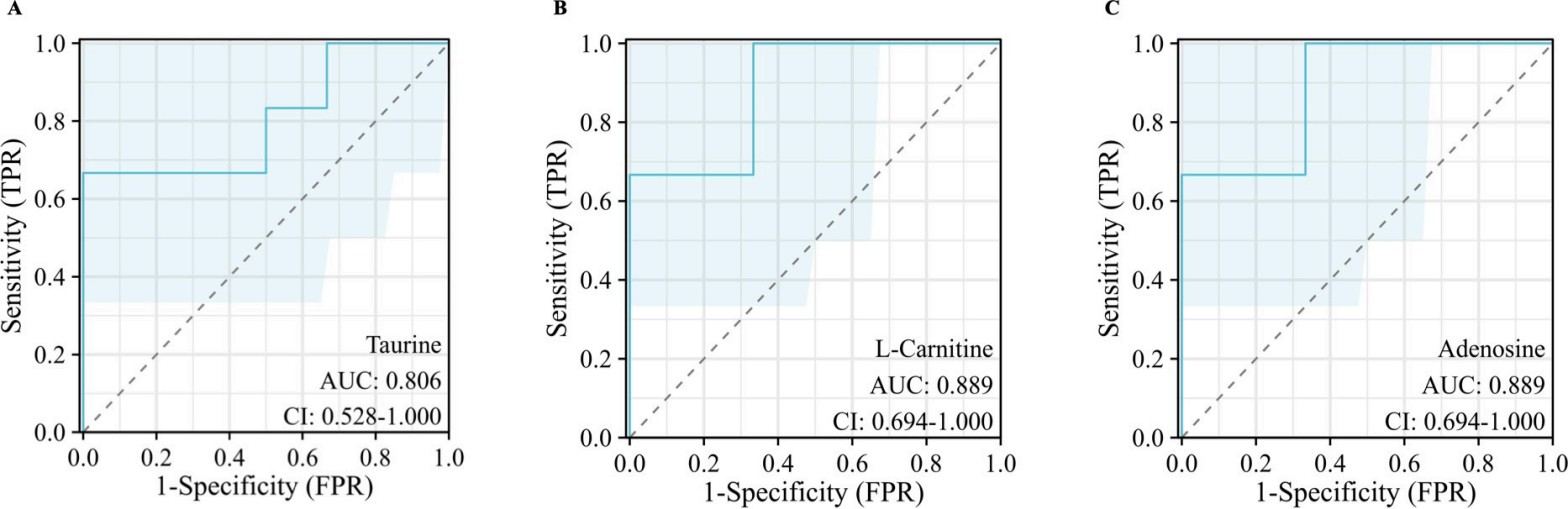
ROC analysis to differentiate TMJ-OA groups from TMJ-DD groups. **(A)** Taurine. **(B)** L-carnitine. **(C)** Adenosine.

**Fig 4:**
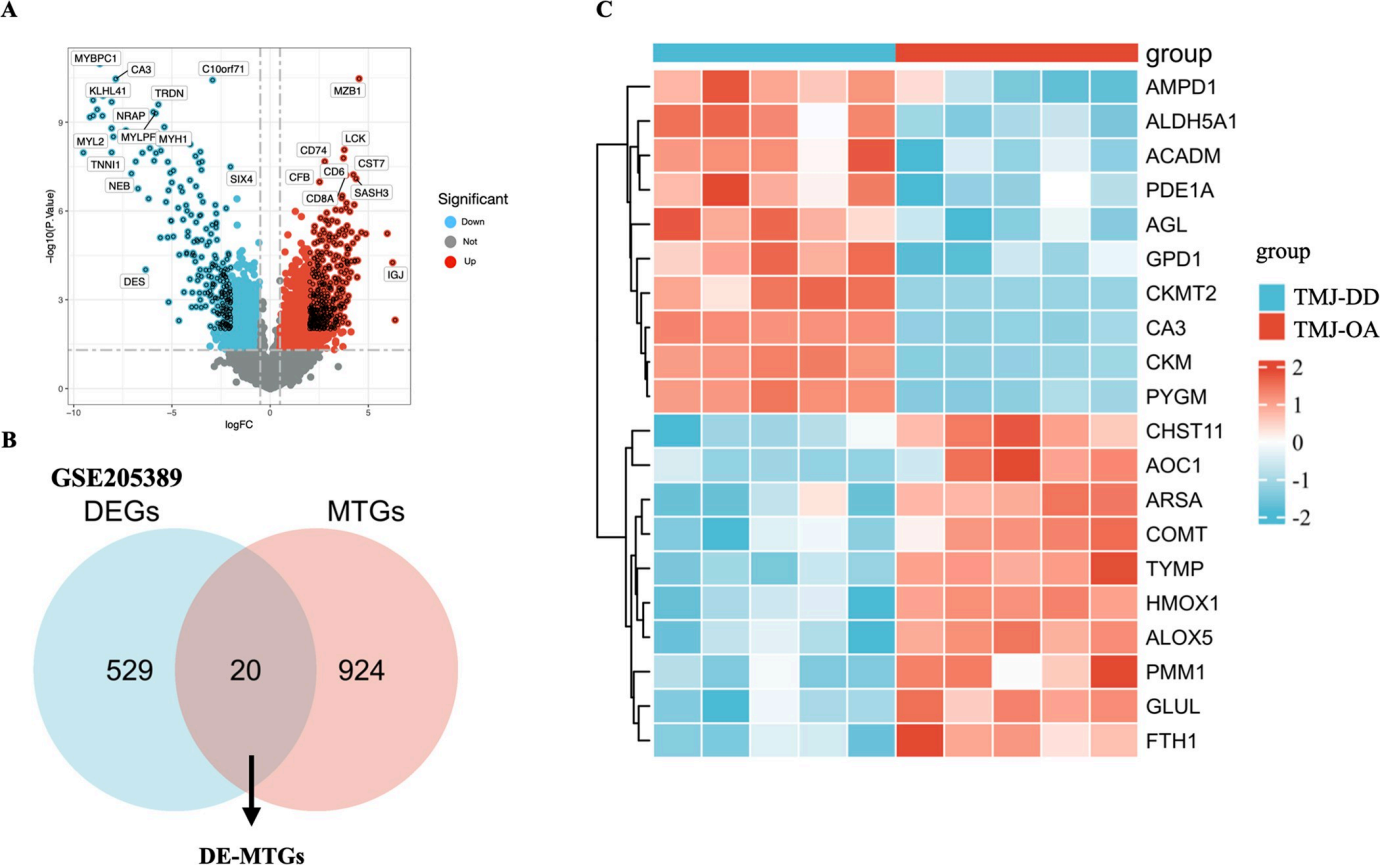
Screening of differentially expressed metabolism-related genes. **(A)** Volcano plot illustrating the 549 DEGs between TMJ-OA and TMJ-DD groups. **(B)** metabolism-related genes (DE-MTGs) in the MSigDB database were intersected with 549 DEGs, and DE-MTGs were selected. **(C)** Heatmap for the 20 DE-MTGs between TMJ-OA and TMJ-DD groups.

**Fig 5:**
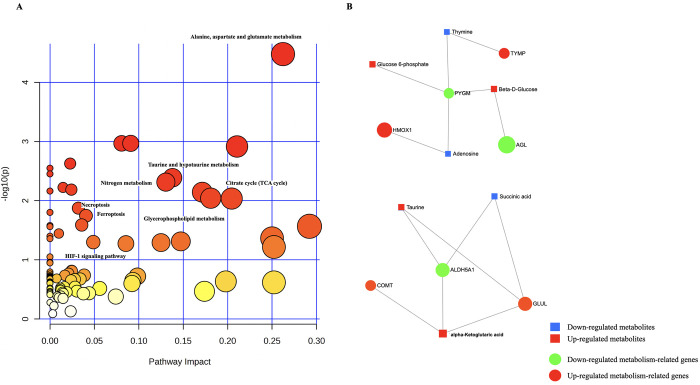
Integrative analysis of metabolomics and transcriptomics. **(A)** Visualization of Integrative pathway analysis results, the size and color of each circle corresponded to the pathway impact value and p-value, respectively. **(B)** network representation of the relationship between DMs and DE-MTGs in metabolomics and transcriptomics analysis.

The captions for Fig 1, Fig 3, Fig 4 and Fig 6 are also missing from the article. The captions have been provided here.

**Fig 6:**
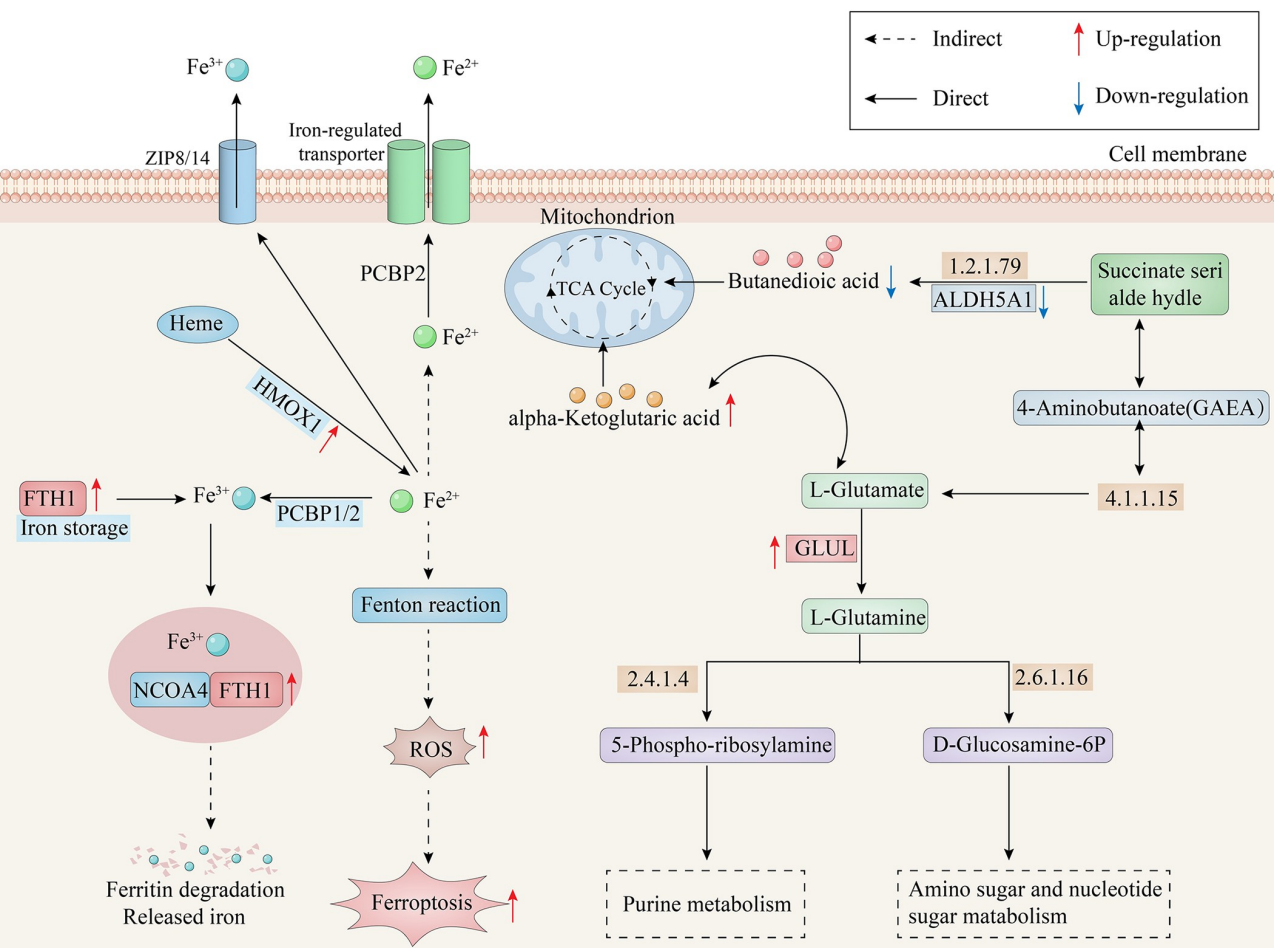
Proposed molecular pathway in TMJ-OA patients

In Table 1, the names of chemical metabolites “Alpha-ketoglutarate (Synonyms: alpha-Ketoglutaric acid)”, “PS(O-20:0/19:1(9Z))” and “PC(18:2(9Z,12Z)/20:2(11Z,14Z))” are omitted and the “Chenodeoxycholic acid” and “Chenodeoxycholic acid glycine conjugate” should be “Butanedioic acid (Synonyms: Succinic acid)” and “Succinylacetone”. Please see the correct Table 1 here.

**Table 1:** Classification of metabolites in synovial fluid based on their chemical compositions.

Identified metabolite	
*Amino acids*	
L-cysteine-glycine	L-Histidinol
*Sugars and sugar alcohols*	
Glycerol-3-galactoside	1-kestose
Melibiose	Beta-D-Glucose
Glucose-6-phosphate	Lactulose
*Organic acids*	
Isohexonic acid	Cerotinic acid
2-furoic acid	Digalacturonic acid
Butanedioic acid (Succinic acid)	2-linoleoyl-sn-glycero-3-phosphocholine
3-amino-2-naphthoic acid	Sulfolithocholylglycine
Alpha-ketoglutarate (alpha-Ketoglutaric acid) Succinylacetone	Tuberonic acid
*Fatty acids*	
LysoPC(18:2(9Z,12Z))	LysoPE (0:0/20:2(11Z,14Z))
SM(d18:1/14:0)	GM4(d18:1/18:0)
SM(d16:1/16:0)	SM(d18:1/16:1)
Palmitelaidic acid	Arachidyl alcohol
Oleic acid	6-Tridecynoic acid
C16 Sphinganine	Lithocholic acid glycine conjugate
PS(O-20:0/19:1(9Z))	PC(18:2(9Z,12Z)/20:2(11Z,14Z))
*Amines*	
Adenosine	Thymine
Cytidine-5-monophosphate	
*Others*	
Octanoate radical	L-acetylcarnitine
L-carnitine	Taurine
Propionylcarnitine	5a-Dihydrotestosterone sulfate
Diethylpropion (metabolite XI Glucuronide)	Avocadene 1-acetate
Piperonyl sulfoxide	P-chlorophenylalanine

In the investigating metabolomic profiles in TMJ-OA and TMJ-DD synovial fluids of subsection of the Results, there is an error in the third sentence. The correct sentence is: The 46 DMs were categorized into different chemical classes in Table 1, including fatty acids (30.4% of the identified metabolites), organic acids (24%), sugars and sugar alcohols (13%), amino acids (4%), amines (7%), and others (21.6%).

In the Discussion, there is an error in the first sentence of the eighth paragraph. The correct sentence is: For the identification of DE-MTGs, we identified 20 DE-MTGs, including 10 upregulated genes (TYMP, HMOX1, ALOX5, GLUL, CHST11, ARSA FTH1, COMT, PMM1, AOC1) and 10 downregulated genes (ACADM, ALDH5A1, AMPD1, CA3, CKMT2, CKM, GPD1, PDE1A, PYGM, AGL).
