# Supplementary material for: Integration of metabolomics and transcriptomics provides insights into the molecular mechanism of temporomandibular joint osteoarthritis
Source: PLoS One. 2024 May 16;19(5):e0301341. doi: 10.1371/journal.pone.0301341 (PMC11098350; doi:10.1371/journal.pone.0301341)
Supplement: S1 Fig — (A) Boxplot of gene probe expression levels among samples. There was no significant difference in the median and the upper and lower quartile. (B) PCA principal-component analysis. (DOCX) [file pone.0301341.s001.docx]

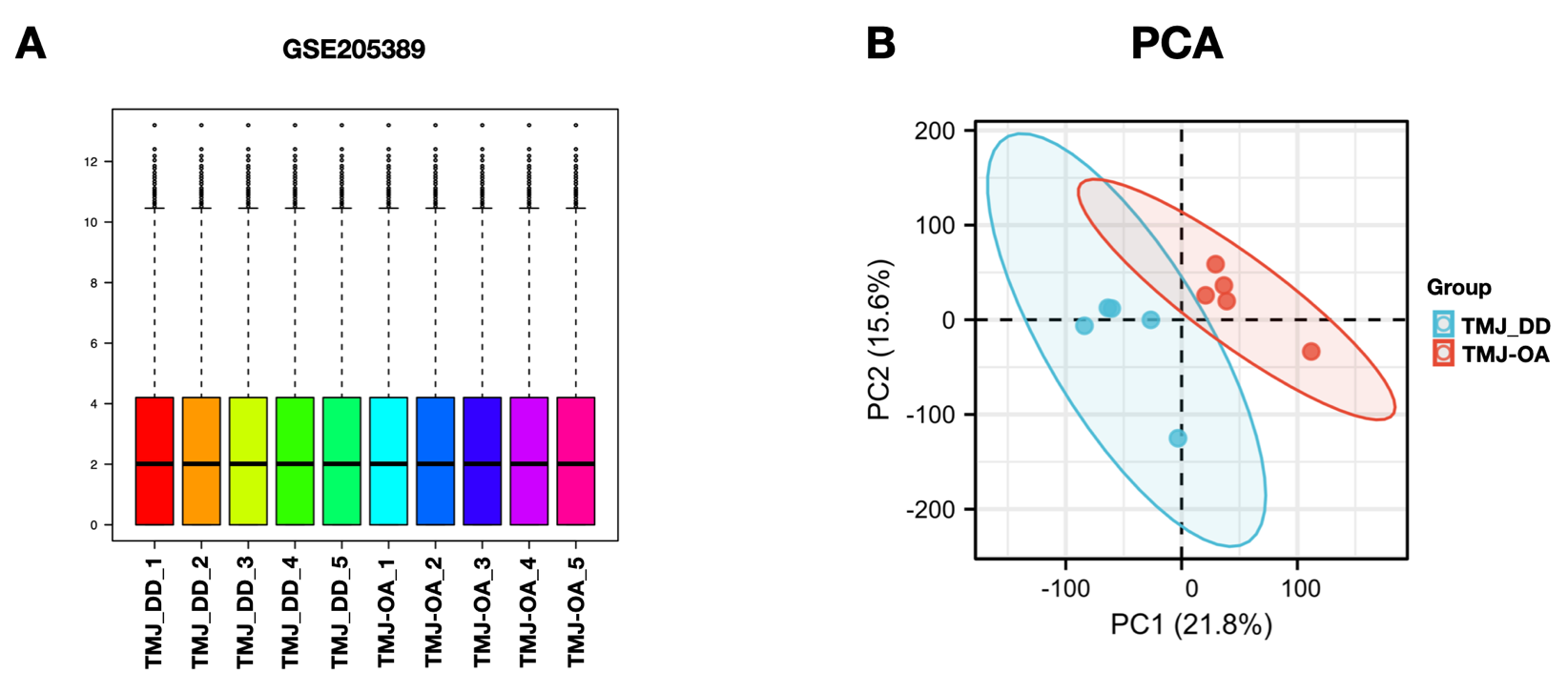


**Fig. S1. (A)** Boxplot of gene probe expression levels among samples. There was no significant difference in the median and the upper and lower quartile. **(B)** PCA principal-component analysis.
