# Supplementary material for: Integration of metabolomics and transcriptomics provides insights into the molecular mechanism of temporomandibular joint osteoarthritis
Source: PLoS One. 2024 May 16;19(5):e0301341. doi: 10.1371/journal.pone.0301341 (PMC11098350; doi:10.1371/journal.pone.0301341)
Supplement: S2 Fig — (A) GO/KEGG analysis of upregulated DE-MTGs. (B) GO/KEGG analysis of downregulated DE-MTGs. (DOCX) [file pone.0301341.s002.docx]

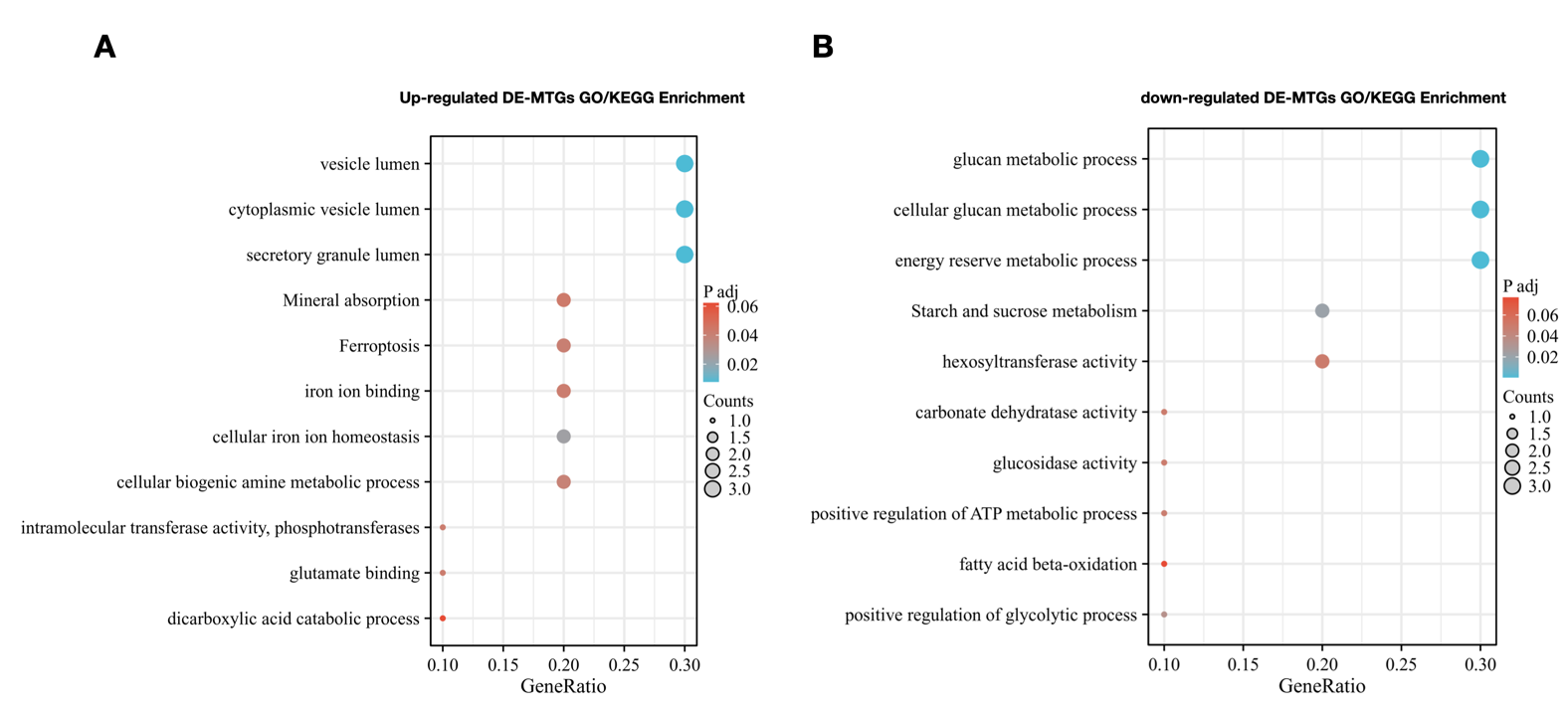


**Fig. S2. Functional enrichment of the DE-MTGs. (A)** GO/KEGG analysis of upregulated DE-MTGs. **(B)** GO/KEGG analysis of downregulated DE-MTGs.
