# Supplementary material for: Integration of metabolomics and transcriptomics provides insights into the molecular mechanism of temporomandibular joint osteoarthritis
Source: PLoS One. 2024 May 16;19(5):e0301341. doi: 10.1371/journal.pone.0301341 (PMC11098350; doi:10.1371/journal.pone.0301341)
Supplement: S1 Table — (DOCX) [file pone.0301341.s003.docx]

Table S1. List of significant metabolites of synovial fluid samples for TMJ-OA patients compared with TMJ-DD people.

| **No** | **ID** | **m/z** | **Retention time (min)** | **Metabolites** | **Compound ID** | **Score** | **Formula** | **Mass Error (ppm)** | **VIP** | **P-value** | **log2(FC)** |
| --- | --- | --- | --- | --- | --- | --- | --- | --- | --- | --- | --- |
| 1 | 595 | 361.2 | 24.913 | 1-kestose | HMDB0011729 | 85.4 |  |  | 1.69976302 | 9.33561E-05 | -1.037527529 |
| 2 | 87 | 125.0875 | 8.005 | 2-furoic acid | HMDB0000617 | 95.1 |  |  | 1.534762855 | 0.000224712 | -0.784654472 |
| 3 | 11.23_564.3307m/z | 564.3307405 | 11.23155 | 2-linoleoyl-sn-glycero-3-phosphocholine | HMDB0062711 | 55.2 | C26H50NO7P | 0.092626686 | 12.30152841 | 0.009160638 | -0.672860391 |
| 4 | 4.05_187.0630n | 205.0967949 | 4.051883333 | 3-amino-2-naphthoic acid | 34509 | 44.3 | C11H9NO2 | -1.895749945 | 5.724613758 | 0.007004063 | -0.522536117 |
| 5 | 8.39_369.1739m/z | 369.1738574 | 8.389966667 | 5a-Dihydrotestosterone sulfate | HMDB0006278 | 39.4 | C19H30O5S | -0.705599045 | 2.448118433 | 0.025043486 | -1.28876479 |
| 6 | 9.14_210.1616n | 228.1954287 | 9.13715 | 6-Tridecynoic acid | 74256 | 52.6 | C13H22O2 | -1.782003075 | 3.168110149 | 0.000144066 | 0.191157528 |
| 7 | 596 | 236.06 | 24.959 | Adenosine | HMDB0000050 | 72.5 |  |  | 2.667662477 | 0.043544447 | -1.881870922 |
| 8 | 537 | 355.3875 | 21.768 | Arachidyl alcohol | HMDB0011619 | 76.6 |  |  | 1.831564562 | 0.000553902 | -1.133496783 |
| 9 | 13.77_329.2694m/z | 329.2693559 | 13.76611667 | Avocadene 1-acetate | LMFA05000640 | 36.9 | C19H36O4 | 2.192833445 | 2.72635351 | 0.004439728 | 0.560299213 |
| 10 | 0.69_180.0629n | 203.052157 | 0.685633333 | Beta-D-Glucose | HMDB0000516 | 45.6 | C6H12O6 | -2.509260739 | 1.605087524 | 0.022571275 | 0.355743709 |
| 11 | 9.17_274.2735m/z | 274.2734775 | 9.168533333 | C16 Sphinganine | 41556 | 48.8 | C16H35NO2 | -2.116124831 | 1.776091617 | 0.009363607 | 0.17712259 |
| 12 | 574 | 325.3125 | 23.682 | Cerotinic acid | HMDB0002356 | 70.3 |  |  | 1.747938834 | 0.002467139 | -1.086790701 |
| 13 | 289 | 243.1 | 14.212 | Cytidine-5-monophosphate |  | 76.1 |  |  | 1.518797044 | 0.000463094 | -0.78678461 |
| 14 | 8.10_416.1561m/z | 416.1561162 | 8.104866667 | Diethylpropion (metabolite XI Glucuronide) | 2025 | 39.7 | C17H25NO8 | -0.278580289 | 3.971002567 | 0.000703599 | 11.55412238 |
| 15 | 388 | 257.1091 | 16.724 | Digalacturonic acid | HMDB0039721 | 83.1 |  |  | 1.577742635 | 0.000170536 | -0.821583257 |
| 16 | 366 | 211 | 16.201 | Glucose-6-phosphate | HMDB0001401 | 64.2 |  |  | 1.385586993 | 0.003790786 | 0.722105242 |
| 17 | 230 | 235.0909 | 12.399 | Glycerol-3-galactoside |  | 80 |  |  | 2.310585041 | 0.027808728 | 2.055378513 |
| 18 | 11.92_1018.6940n | 1019.701279 | 11.9221 | GM4(d18:1/18:0) | LMSP0601AA02 | 38.1 | C53H98N2O16 | 2.323971982 | 1.923774324 | 0.020953797 | 0.244076043 |
| 19 | 520 | 311.2222 | 20.888 | Isohexonic acid |  | 83.1 |  |  | 2.175331814 | 0.037212747 | 1.535483204 |
| 20 | 0.77_203.1153n | 204.1226689 | 0.7689 | L-Acetylcarnitine | HMDB0000201 | 55.3 | C9H17NO4 | -2.009926726 | 5.340319543 | 0.038816888 | 0.519389954 |
| 21 | 335 | 255.1 | 15.568 | Lactulose | HMDB0000740 | 75.8 |  |  | 1.098286294 | 0.00583757 | -0.522863652 |
| 22 | 0.69_162.1121m/z | 162.1121451 | 0.685633333 | L-Carnitine | 34532 | 56.3 | C7H15NO3 | -2.01533274 | 4.210660809 | 0.018890735 | 0.291349444 |
| 23 | 363 | 257.1 | 16.097 | L-cysteine-glycine |  | 79.9 |  |  | 1.542888763 | 0.000413778 | -0.812581042 |
| 24 | 15.81_124.0869m/z | 124.0868865 | 15.81448333 | L-Histidinol | 301 | 38.5 | C6H11N3O | -0.263678389 | 2.424772745 | 0.017841134 | 0.186215132 |
| 25 | 9.68_433.3184n | 416.3151568 | 9.681483333 | Lithocholic acid glycine conjugate | HMDB0000698 | 57.9 | C26H43NO4 | -1.762789278 | 1.679152729 | 0.016884563 | -1.996792598 |
| 26 | 11.23_519.3317n | 520.339008 | 11.22871667 | LysoPC(18:2(9Z,12Z)) | HMDB0010386 | 58.1 | C26H50NO7P | -1.459551551 | 23.36937625 | 0.025749511 | -0.507056313 |
| 27 | 11.23_504.3101m/z | 504.3100803 | 11.23155 | LysoPE(0:0/20:2(11Z,14Z)) | 62271 | 38.7 | C25H48NO7P | 1.023614434 | 1.679469311 | 0.00982192 | -0.734076685 |
| 28 | 600 | 204.1 | 25.275 | Melibiose | HMDB0000048 | 94.3 |  |  | 1.624150515 | 0.003917472 | -0.979126884 |
| 29 | 163 | 201.1 | 10.199 | Octanoate radical |  | 97.9 |  |  | 1.986024129 | 0.015153579 | 1.386416146 |
| 30 | 554 | 129.08 | 22.534 | Oleic acid | HMDB0000207 | 99.5 |  |  | 1.527119473 | 0.024537584 | 0.957276126 |
| 31 | 521 | 117.05 | 20.942 | Palmitelaidic acid | HMDB0012328 | 98.8 |  |  | 2.092172827 | 0.027407749 | 1.666340182 |
| 32 | 14.45_832.5814m/z | 832.5814415 | 14.4501 | PC(18:2(9Z,12Z)/20:2(11Z,14Z)) | LMGP01011632 | 36.2 | C46H84NO8P | -1.52482574 | 7.90683497 | 0.003644334 | 0.746225698 |
| 33 | 4.30_200.0470m/z | 200.0469676 | 4.296833333 | p-CHLOROPHENYLALANINE | 44308 | 52.5 | C9H10ClNO2 | -1.583318513 | 2.89218885 | 0.006583691 | 0.294497917 |
| 34 | 8.73_369.1739m/z | 369.173851 | 8.728533333 | Piperonyl sulfoxide | 72888 | 39.3 | C18H28O3S | -0.825551436 | 5.139709444 | 0.006835746 | -1.260532034 |
| 35 | 1.35_218.1383m/z | 218.1382577 | 1.348416667 | Propionylcarnitine | HMDB0000824 | 49.4 | C10H19NO4 | -1.965682973 | 1.874345187 | 0.025564198 | -0.73668063 |
| 36 | 14.45_856.5800m/z | 856.5800313 | 14.4501 | PS(O-20:0/19:1(9Z)) | 78705 | 37.1 | C45H88NO9P | -3.420723191 | 3.335012064 | 0.020668511 | 1.051978236 |
| 37 | 13.75_675.5422m/z | 675.5422355 | 13.75141667 | SM(d16:1/16:0) | 83737 | 38.7 | C37H75N2O6P | -1.950453751 | 11.38522977 | 0.00158924 | 0.583677036 |
| 38 | 13.70_719.5351m/z | 719.5350928 | 13.6992 | SM(d18:1/14:0) | HMDB0012097 | 56.7 | C37H75N2O6P | 0.911979575 | 2.289908755 | 0.017050724 | 0.575552703 |
| 39 | 14.07_745.5507m/z | 745.5506636 | 14.07158333 | SM(d18:1/16:1) | LMSP03010041 | 37.6 | C39H77N2O6P | 0.764947972 | 2.143018544 | 0.009480308 | 0.412238825 |
| 40 | 593 | 257.1 | 24.877 | Succinylacetone | HMDB0000635 | 79.4 |  |  | 1.688068338 | 0.001638198 | -0.995990867 |
| 41 | 9.68_512.2686m/z | 512.2685812 | 9.683333333 | Sulfolithocholylglycine | HMDB0002639 | 50.2 | C26H43NO7S | -0.323591206 | 2.990414079 | 0.015887545 | -2.160739136 |
| 42 | 379 | 326.12 | 16.474 | Taurine | HMDB0000251 | 96.3 |  |  | 1.329388802 | 0.042977213 | 1.070774273 |
| 43 | 262 | 255.1 | 13.477 | Thymine | HMDB0000262 | 88.8 |  |  | 1.587689762 | 0.000217482 | -0.842756627 |
| 44 | 9.05_207.1018m/z | 207.1018495 | 9.050916667 | Tuberonic acid | LMFA02020007 | 45.3 | C12H18O4 | -3.619661117 | 1.524571811 | 0.000847257 | 1.757146955 |
| 45 | 323 | 198.0667 | 15.158 | Alpha-ketoglutarate | HMDB0000208 | 85.7 |  |  | 1.180000868 | 0.017306788 | 0.620190789 |
| 46 | 191 | 147.1 | 11.028 | Butanedioic acid | HMDB0000254 | 94.9 |  |  | 1.429025037 | 0.000271377 | -0.690935419 |
