# Supplementary material for: Integration of metabolomics and transcriptomics provides insights into the molecular mechanism of temporomandibular joint osteoarthritis
Source: PLoS One. 2024 May 16;19(5):e0301341. doi: 10.1371/journal.pone.0301341 (PMC11098350; doi:10.1371/journal.pone.0301341)
Supplement: S4 Table — (DOCX) [file pone.0301341.s006.docx]

| **ID** | **logFC** | **AveExpr** | **t** | **P.Value** | **adj.P.Val** | **B** |
| --- | --- | --- | --- | --- | --- | --- |
| TYMP | 2.764377 | 4.3350691 | 10.073284 | 5.50E-06 | 0.00104972 | 4.61429223 |
| HMOX1 | 2.491017 | 4.1037853 | 7.214852 | 7.01E-05 | 0.00665459 | 1.9489077 |
| ALOX5 | 3.88686 | 3.5486807 | 6.867967 | 0.00010047 | 0.00839984 | 1.56697839 |
| GLUL | 1.032081 | 8.3459629 | 6.67611 | 0.00012335 | 0.00978718 | 1.34937644 |
| CHST11 | 1.986304 | 3.4590558 | 5.584 | 0.00043184 | 0.02293268 | 0.01827917 |
| ARSA | 2.914546 | 2.2328706 | 5.307985 | 0.00060754 | 0.02828515 | -0.3444239 |
| FTH1 | 1.037991 | 11.3015389 | 5.213449 | 0.00068456 | 0.03004469 | -0.4711819 |
| COMT | 1.700511 | 5.1482027 | 5.066841 | 0.00082581 | 0.03438715 | -0.6703336 |
| PMM1 | 2.648473 | 1.7933262 | 5.012012 | 0.00088652 | 0.03623646 | -0.7456186 |
| AOC1 | 2.36516 | 1.3716391 | 4.693004 | 0.00135113 | 0.04787649 | -1.1923289 |
| ACADM | -1.5430781 | 5.15762254 | -5.2353286 | 0.00066583 | 0.02981456 | -0.4417297 |
| ALDH5A1 | -2.3694994 | 1.59392842 | -6.8032293 | 0.00010762 | 0.0088157 | 1.49406967 |
| AMPD1 | -2.9414587 | 2.39562283 | -4.83244 | 0.00112181 | 0.04295611 | -0.9952545 |
| CA3 | -7.8593335 | 4.22413354 | -43.204582 | 3.43E-11 | 1.92E-07 | 14.4490321 |
| CKMT2 | -4.9276944 | 2.46485332 | -9.7043699 | 7.36E-06 | 0.00127335 | 4.31228851 |
| COMT | 1.70051083 | 5.14820271 | 5.06684127 | 0.00082581 | 0.03438715 | -0.6703336 |
| GPD1 | -2.9147617 | 2.36157479 | -5.9439902 | 0.00028098 | 0.01724435 | 0.4750363 |
| PDE1A | -1.986538 | 3.83062819 | -4.731913 | 0.00128243 | 0.04685687 | -1.1370525 |
| PYGM | -5.5625919 | 3.32296869 | -22.119915 | 9.23E-09 | 7.45E-06 | 10.6991702 |
| AGL | -2.2165449 | 3.92996636 | -5.7480213 | 0.00035429 | 0.01974726 | 0.22865209 |

**Table S4. The 20 DE-MTGs in TMJ-OA human synovial membrane**
