# Supplementary material for: Integration of metabolomics and transcriptomics provides insights into the molecular mechanism of temporomandibular joint osteoarthritis
Source: PLoS One. 2024 May 16;19(5):e0301341. doi: 10.1371/journal.pone.0301341 (PMC11098350; doi:10.1371/journal.pone.0301341)
Supplement: S5 Table — (DOCX) [file pone.0301341.s007.docx]

**Table S5. KEGG pathways enriched by Integration analysis of metabolomics and transcriptomics.**

| **KEGG metabolic pathways** | **Expected** | **Raw p** | **#NAME?** | **FDR** | **Impact** | **matched_features** |
| --- | --- | --- | --- | --- | --- | --- |
| Alanine, aspartate and glutamate metabolism | 0.18611 | 3.33E-05 | 4.4776 | 0.011021 | 0.2623 | cpd:C00042; cpd:C00026; hsa:7915; hsa:2752 |
| Histidine metabolism | 0.20356 | 0.0010741 | 2.969 | 0.10039 | 0.080645 | hsa:26; cpd:C00860; cpd:C00026 |
| Butanoate metabolism | 0.20356 | 0.0010741 | 2.969 | 0.10039 | 0.090909 | hsa:7915; cpd:C00042; cpd:C00026 |
| Starch and sucrose metabolism | 0.21228 | 0.0012132 | 2.9161 | 0.10039 | 0.21053 | cpd:C00092; hsa:178; hsa:5837 |
| Glyoxylate and dicarboxylate metabolism | 0.26754 | 0.0023593 | 2.6272 | 0.15578 | 0.022727 | cpd:C00042; cpd:C00026; hsa:2752 |
| GABAergic synapse | 0.28498 | 0.0028239 | 2.5492 | 0.15578 | 0 | hsa:2752; cpd:C00026; cpd:C00042 |
| Central carbon metabolism in cancer | 0.30825 | 0.0035258 | 2.4527 | 0.16672 | 0 | cpd:C00092; cpd:C00026; cpd:C00042 |
| Taurine and hypotaurine metabolism | 0.095964 | 0.0040927 | 2.388 | 0.16934 | 0.13793 | cpd:C00245; cpd:C00026 |
| Nitrogen metabolism | 0.10469 | 0.0048569 | 2.3136 | 0.17862 | 0.13043 | hsa:761; hsa:2752 |
| Insulin resistance | 0.37222 | 0.0059743 | 2.2237 | 0.18302 | 0.014706 | cpd:C02571; hsa:5837; cpd:C00092 |
| Glucagon signaling pathway | 0.38386 | 0.0065053 | 2.1867 | 0.18302 | 0.024096 | cpd:C00026; cpd:C00042; hsa:5837 |
| Sulfur metabolism | 0.12504 | 0.0068738 | 2.1628 | 0.18302 | 0 | cpd:C00042; cpd:C00245 |
| Arginine biosynthesis | 0.12795 | 0.0071881 | 2.1434 | 0.18302 | 0.17143 | hsa:2752; cpd:C00026 |
| Glycerophospholipid metabolism | 0.43329 | 0.0090725 | 2.0423 | 0.20318 | 0.18095 | hsa:2819; cpd:C04230; cpd:C00157 |
| Citrate cycle (TCA cycle) | 0.1454 | 0.0092075 | 2.0359 | 0.20318 | 0.20455 | cpd:C00042; cpd:C00026 |
| Necroptosis | 0.50018 | 0.013376 | 1.8737 | 0.27671 | 0.031915 | hsa:5837; hsa:2752; hsa:2495 |
| ABC transporters | 0.53216 | 0.015783 | 1.8018 | 0.3073 | 0 | cpd:C00245; cpd:C02273; cpd:C00212 |
| Ferroptosis | 0.20647 | 0.017983 | 1.7451 | 0.33069 | 0.040541 | hsa:2495; hsa:3162 |
| Renin secretion | 0.25009 | 0.025745 | 1.5893 | 0.41278 | 0.035714 | cpd:C00212; hsa:5136 |
| Mineral absorption | 0.253 | 0.026304 | 1.58 | 0.41278 | 0 | hsa:2495; hsa:3162 |
| Purine metabolism | 0.6543 | 0.027104 | 1.567 | 0.41278 | 0.29208 | hsa:5136; hsa:270; cpd:C00212 |
| Pentose and glucuronate interconversions | 0.25881 | 0.027436 | 1.5617 | 0.41278 | 0 | cpd:C02273; cpd:C00026 |
| Morphine addiction | 0.29952 | 0.03589 | 1.445 | 0.5165 | 0.01 | cpd:C00212; hsa:5136 |
| Choline metabolism in cancer | 0.31697 | 0.039785 | 1.4003 | 0.54869 | 0 | cpd:C00157; cpd:C04230 |
| Tyrosine metabolism | 0.33151 | 0.043148 | 1.365 | 0.55803 | 0.25 | cpd:C00042; hsa:1312 |
| Lysine degradation | 0.33442 | 0.043833 | 1.3582 | 0.55803 | 0 | cpd:C00026; cpd:C00042 |
| Pyrimidine metabolism | 0.35478 | 0.048743 | 1.3121 | 0.58333 | 0.14729 | hsa:1890; cpd:C00178 |
| HIF-1 signaling pathway | 0.36059 | 0.050182 | 1.2995 | 0.58333 | 0.04878 | hsa:3162; cpd:C00026 |

Note: It was revealed by integration analysis of metabolomics and transcriptomics that 28 KEGG pathways were with significance alterations
